# Synthesis of the 1,5-disubstituted tetrazole-methanesulfonylindole hybrid system via high-order multicomponent reaction

**DOI:** 10.3762/bjoc.20.256

**Published:** 2024-11-26

**Authors:** Cesia M Aguilar-Morales, América A Frías-López, Nadia V Emilio-Velázquez, Alejandro Islas-Jácome, Angelica Judith Granados-López, Jorge Gustavo Araujo-Huitrado, Yamilé López-Hernández, Hiram Hernández-López, Luis Chacón-García, Jesús Adrián López, Carlos Jesús Cortés-García

**Affiliations:** 1 Laboratorio de Diseño Molecular, Instituto de Investigaciones Químico-Biológicas, Universidad Michoacana de San Nicolás de Hidalgo. Ciudad Universitaria, C.P. 58030, Morelia, Michoacán, Mexico; 2 Departamento de Química, Universidad Autónoma Metropolitana-Iztapalapa, Av. Ferrocarril San Rafael Atlixco 186, Col. Leyes de Reforma 1A Sección, Iztapalapa, 09310, Ciudad de México, Mexico; 3 Laboratorio de microRNAs y Cáncer, Universidad Autónoma de Zacatecas, Av. Preparatoria S/N, Agronómica, Campus II, Zacatecas 98066, Zacatecas, Mexico; 4 Laboratorio de Metabolómica y Proteómica, Cátedra CONACYT, Unidad Académica de Ciencias Biológicas, Universidad Autónoma de Zacatecas, Av. Preparatoria S/N, Agronómica, Campus II, Zacatecas 98066, Zacatecas, Mexico; 5 Laboratorio de síntesis y modificación química, Unidad Académica de Ciencias Químicas, Universidad Autónoma de Zacatecas, Campus UAZ siglo XXI, carretera Zacatecas-Guadalajara km 6, Zacatecas, Zacatecas, 98160, México

**Keywords:** 1,5-disubstituted tetrazoles, high-order multicomponent reaction, isocyanides, MCF-7 cell line, methanesulfonylindoles, Ugi-azide reaction

## Abstract

A series of 1,5-disubstituted tetrazole-indole hybrids were synthesized via a high-order multicomponent reaction consisting of an Ugi-azide/Pd/Cu-catalyzed hetero-annulation cascade sequence. This operationally simple one-pot protocol allowed high bond-forming efficiency and creating six new bonds (two C–C, three C–N, and one N–N). Additionally, the products were evaluated against breast cancer MCF-7 cells, finding moderate activity in the compounds substituted with fluorine and chlorine.

## Introduction

Nitrogen-containing heterocyclic moieties, such as 1,5-disubstituted tetrazoles and indoles, are considered pharmacophoric fragments due to their pivotal interactions with several targets involved in many diseases. They are found in a plethora of compounds with significant biological and pharmacological relevance [1–4]. These moieties are indispensable in the design and synthesis of novel drugs aimed at overcoming drug resistance, which is a global health threat [5–7]. To date, a strategy to access novel highly bioactive compounds that can be converted into drug candidates in a fast and efficient manner containing these moieties is the molecular hybridization [8–10]. It consists of covalently joining of two or more pharmacophoric fragments to provide new hybrid compounds with improved efficacy and affinity compared to their drug parents and by using powerful synthetic tools such as multicomponent reactions (MCRs) [11–13]. Among these, isocyanide-based multicomponent reactions (I-MCRs), such as the Ugi-azide reaction, have demonstrated the highest biological-synthetic relevance [1,14–16]. In this regard, a relatively unexplored field within MCRs comprises high-order multicomponent reactions, which involve reacting at least five or even more components in a single operational step. These reactions are highly convergent, efficient, and have superior atom economy, producing high structural diversity and complexity compared to classical 3- or 4-CRs [17–19].

**Scheme 1:**
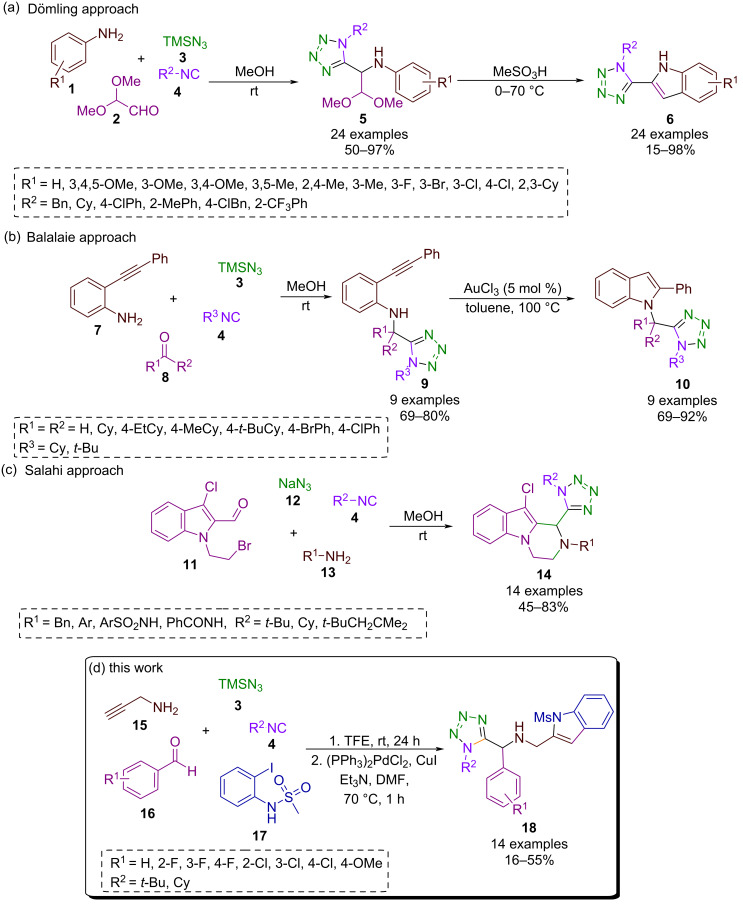
Synthetic approaches to obtain the 1,5-disubstituted tetrazole-indole system and our synthetic approach.

In the same context, the hybridization of 1,5-disubstituted tetrazoles with an indole moiety using I-MCRs such as the Ugi-azide reaction as a key step, is very limited. To our knowledge, only three reactions have been described (Scheme 1a–c). In 2021, Dömling’s research group synthesized a series of 1,5-disubstituted tetrazole-indoles **6** in good to excellent yields via an Ugi-azide/acidic ring-closure sequence [20]. Balalaie described an efficient method in 2018 for the synthesis of a new 1,5-disubstituted tetrazole-indole system **10**, in a two-step reaction: Ugi-azide followed by a cyclization reaction catalyzed by AuCl_3_, in good to high yields [21]. In 2019, Salahi et al. synthesized the series of tetrazole-indoles **15** via an Ugi-azide reaction in moderate to high yields [22]. It is noteworthy that none of the previously described strategies involved the use of higher-order multicomponent reactions (HO-MCRs).

As part of our ongoing research program towards the synthesis of novel hybrid compounds based on the 1,5-disubstituted tetrazole moiety [23–27], we developed a synthetic strategy for the synthesis of a novel bis-heterocyclic hybrid, 1,5-disubstituted-tetrazole-indoles. The compounds were achieved through a high-order multicomponent reaction consisting of two sequential processes: an Ugi-azide reaction and a further Pd/Cu-catalyzed heteroannulation (Scheme 1d).

## Results and Discussion

**Scheme 2:**
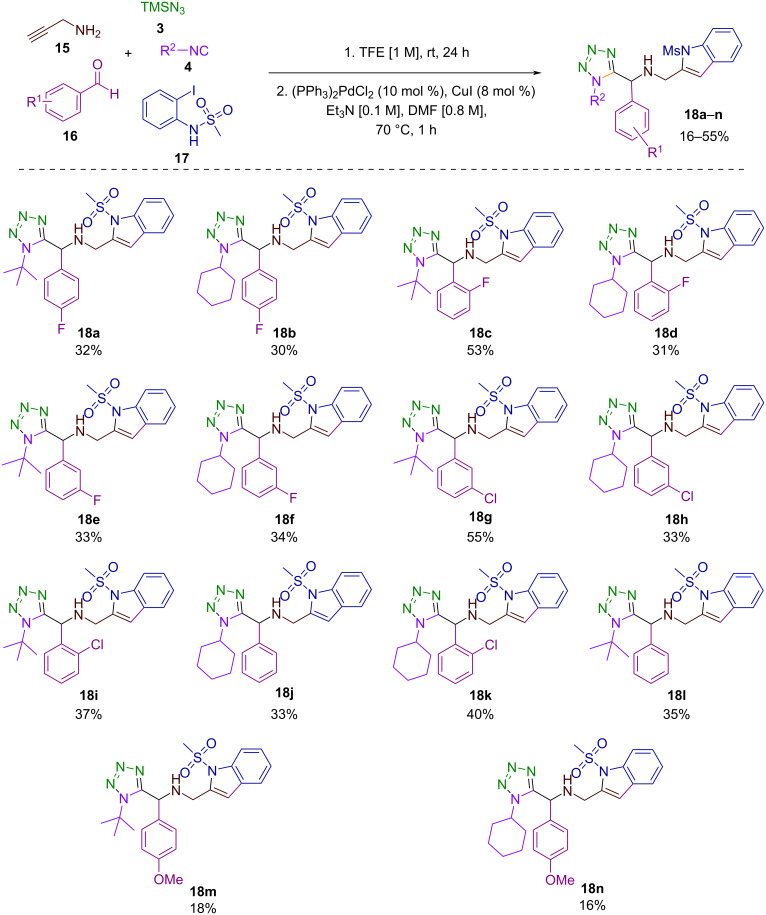
High-order multicomponent reaction for the synthesis of 1,5-disubstituted tetrazol-methanesulfonylindole hybrids.

Our study commenced with synthesizing a series of 1,5-disubstituted tetrazole-methanesulfonylindoles **18a**–**n**, achieved through a high-order multicomponent approach. This strategy, similar to that previously reported by our research group for synthesizing the 1,5-disubstituted tetrazole-benzofuran bis-heterocyclic system [23], involves a two-step sequence starting with an Ugi-azide multicomponent reaction, followed by a Pd/Cu-catalyzed heteroannulation process, as depicted in Scheme 2. Conditions for the Ugi-azide reaction were optimized based on our recent findings, i.e., the reaction was performed in trifluoroethanol as the solvent and at room temperature [23–26]. In the initial reaction, propargylamine served as a bifunctional reagent, with the primary amine group participating in the first step and the terminal alkyne promoting the subsequent heteroannulation. (Scheme 2). As observed in our previous studies, we employed benzaldehyde derivatives with various stereoelectronic decoration, excluding aliphatic ones due to their decreased reactivity resulting in failures to proceed [24–27]. Commercially available isocyanides utilized were cyclohexyl and *tert*-butyl isocyanide. Notably, an isolation of the 1,5-disubstituted tetrazole-alkyne intermediates was unnecessary; we previously reported this heterocyclic system [24–26]. The heteroannulation reaction was then investigated under the most common conditions, utilizing a catalytic system comprising PdCl_2_(PPh_3_)_2_, Et_3_N and CuI [23,28]. Therefore, the key component for this reaction was methanesulfonyl 2-iodoaniline **17**, as it has been reported that the use of 2-iodoaniline results in the formation of only the Sonogashira coupling product [29–30]. Moreover, as shown in Scheme 2, this high-order multicomponent protocol yielded fourteen 1,5-disubstituted tetrazole-methanesulfonylindole derivatives, with yields ranging from 16% to 55% after purification by column chromatography. Despite some yields being modest, they are deemed reasonable considering the reaction’s structural complexity, atom economy, and the efficiency achieved in terms of time and resource optimization. In addition, all the target compounds were fully characterized using ^1^H and ^13^C NMR spectroscopy and HRMS. It is important to mention that this protocol cannot be considered a true one-pot synthesis, as it requires a solvent exchange between reaction steps (e.g., from trifluoroethanol to Et_3_N for the subsequent catalysis). Thus, this protocol enabled a straightforward and rapid synthesis of highly 2-substituted indoles under mild reaction conditions, highlighting the versatility of propargylamine as a bifunctional reagent in post-Ugi-azide transformations. Our group pioneered the application of this reagent as a central building block in the synthesis of hybrid systems via Ugi-azide reactions, including structures based on triazoles-tetrazoles [24,26–27], benzofuran-tetrazoles [23] and indolizines-tetrazoles [25], all of which have significant relevance in medicinal chemistry and optical science.

**Scheme 3:**
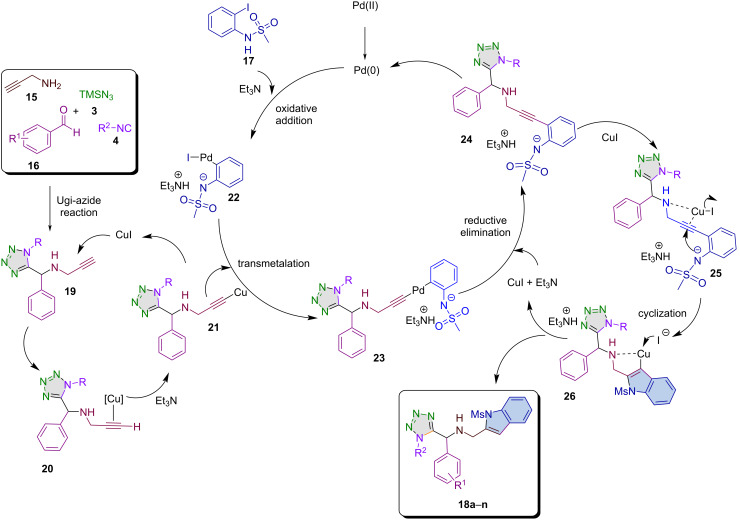
Plausible reaction mechanism for the synthesis of target molecules **18a**–**n**.

A plausible reaction mechanism for the formation of the target molecules **18a**–**n** via a high-order multicomponent reaction is shown in Scheme 3 and consists of two processes: an Ugi-azide reaction and a Pd/Cu-catalyzed heteroannulation reaction. The Ugi-azide reaction mechanism to obtain the 1,5-disubstituted tetrazole-alkyne **19** is well-documented and hence, it is not herein described in detail [1,26,31]. Thus, based on Pal and co-workers’ proposal [32–33], the second process involves two catalytic cycles: 1) a Sonogashira coupling, and 2) a 5-*endo*-*dig* cyclization. The first catalytic cycle begins with the coupling of 1,5-disubstituted tetrazole-alkyne **19** and methanesulfonyl-2-iodoaniline **17** forming the intermediate **23**. Following a reductive elimination, the Sonogashira-like product **24** is produced, which then progresses into the second catalytic cycle. In this cycle, an intramolecular cyclization takes place, facilitated by CuI. This step involves a 5-*endo*-*dig* cyclization, where the negatively nitrogen atom of the sulfonamide **25** attacks intramolecularly to yield the intermediate **26**. The final product is formed when iodide is regenerated as CuI, allowing it to re-enter into the catalytic cycle.

On the other hand, the sulfonyl group in its sulfonamide form is typically associated with antibacterial activity. However, it has been little studied the sulfonyl group regarding biological activity when attached to the indole nitrogen. Although scarce, some recent representative studies include antibacterial, anti-inflammatory, antioxidant, selective inhibitor of COX-2 [34], and anti-HIV activity [35]. In this context, even though indole is considered a privileged scaffold present in some anticancer agents [36], a few examples of methanesulfonylindoles are studied as cytotoxic compounds. For example, a cytotoxic effect was found in CAL 27 cells, presumably through a mechanism of TNF-α inhibition in vitro, which could be related to the anti-inflammatory effect identified in several studies of NSAIDs that inhibit cancer cell viability in vitro [37–38]. In another study aimed at inhibiting tubulin assembly, *N*-mesyl-2-(1-phenylvinyl)indoles were active against HCT-116 cells on the order of a GI_50_ of 10 mM, although the mechanism originally sought was not demonstrated [39].

**Figure 1:**
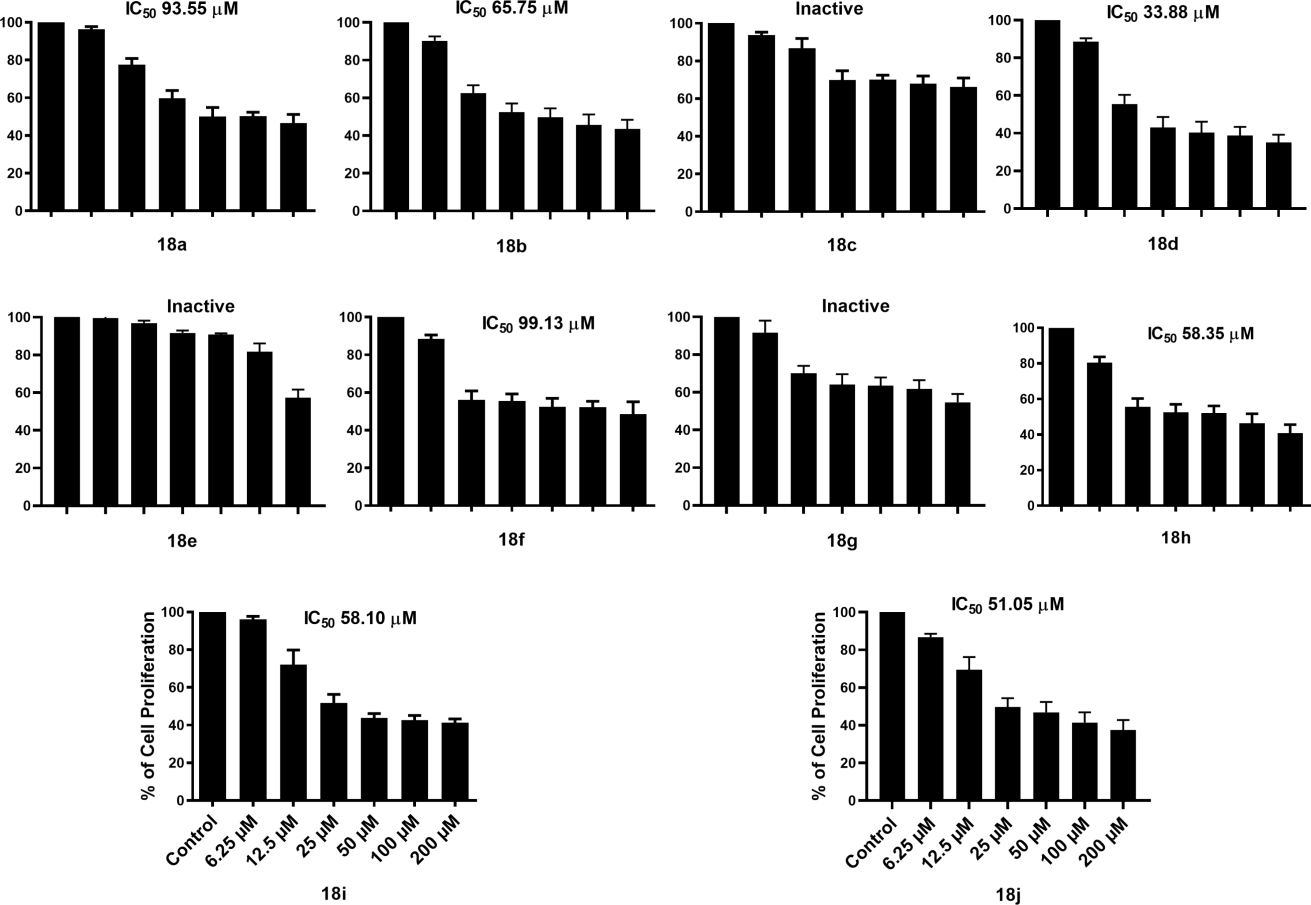
Differential effect of the 1,5-disubstituted tetrazole-indole hybrid compounds **18a**–**j** on proliferation of MCF-7 cell line. MCF-7 cells were treated with increasing doses of compounds **18a**–**j**. Controls were cells treated with DMSO.

Therefore, in this work, the cytotoxic activity of methanesulfonylindoles **18a**–**j** was explored against MCF-7 cell line, which has been used as a model for breast cancer (BC), a public health issue very common and a deadly pathology worldwide, where women between 45 and 55 years of age are the most vulnerable population. In 2020, 684,996 deaths were recorded [40–43]. This in vitro bioactivity study was proposed based on observations that indole-coumarin-thiadiazole hybrid compounds described by Kamath et al. [44], as well as indole-benzimidazole hybrids reported by Singla et al*.* [45] have shown potential as therapeutic agents for breast cancer treatment. Thereby, we hypothesized that combining the indole moiety with the 1,5-disusbtituted tetrazole pharmacophore could increase the non-covalent interactions, including π–π stacking, hydrogen bonding, and hydrophobic interactions. This combination may improve the pharmacodynamic profile, providing a solid foundation for developing compounds with greater efficacy and selectivity against targets related to BC. Thus, the 1,5-disubstituted tetrazole-indole hybrids, presented different effects on cell proliferation inhibition in MCF-7 cells (Figure 1) that could be attributable to the molecular background of cells [46–49]. The compounds that elicited proliferation inhibition are ordered below from the highest to lowest effect on MCF-7 cell line: **18d**, **18j**, **18h**, **18i**, **18b**, **18f**, **18a**, **18g**, **18c**, and **18e**. Compounds **18c**, and **18e** did not affect MCF-7 cell proliferation inhibition. Regarding the structure–activity relationship, the importance of the *tert*-butyl and cyclohexyl substituents in the imidazole can be deduced. According to the IC_50_ results presented in Figure 1, it can be seen that all cyclohexyl-substituted derivatives tested show activity. In contrast, those carrying the *tert*-butyl group are inactive, except for **18i** and **18a**, which show moderate and low activity, respectively. The modification of this substituent is seen when comparing the analogous derivatives **18d** and **18c**; **18h** and **18g**; **18b** and **18a**; **18f** and **18e**, where the replacement of cyclohexyl by *tert*-butyl leads to a loss of activity in each case. Concerning the substituent on the phenyl group, it is interesting to note that the most active compound of the whole series is the fluorinated derivative **18d**, followed in order of inhibition by **18j**, which has no substituents. The position of this halogen is also relevant since the activity decreases when it is in the 4' position (**18b**) and is almost lost when it is in the 3' position (**18a**). Interestingly, compound **18i**, which contains chlorine at the 2'-position, is the only derivative with significant activity in the series of *tert*-butyl derivatives. This suggests that a chloro or fluoro substituent at this position is relevant for the cytotoxic activity.

The 1,5-disubstituted tetrazole-1,2,3-triazole hybrids synthesized by our group [26] had similar effects to the present compounds, suggesting that 1,5-disubstituted tetrazole and indole are pharmacophoric fragments with significant biological and pharmacological potential in anticancer drug design. However, it should be taken into account that the sulfonyl group can radically change the pharmacokinetic properties of a drug, so the effect it may have on its ADMET properties should be determined in an additional study.

## Conclusion

A novel synthetic strategy has been developed for synthesizing a small series of 1,5-disubstituted tetrazole-methanesulfonylindole hybrid compounds under mild reaction conditions. This strategy employs a cascade process consisting of a sequential Ugi-azide and Pd/Cu-catalyzed heteroannulation reactions, achieving low to moderate yields. Significantly, this protocol enables the rapid and straightforward synthesis of highly 2-substituted indoles with high bond-forming efficiency, creating six new bonds (two C–C, three C–N, and one N–N). Our synthetic strategy would represent the second report in which the Pd/Cu-catalyzed heteroannulation reaction is utilized as a post-Ugi-azide reaction, using propargylamine as a key bifunctional reagent. The results of cytotoxic activity were moderate. However, the information obtained from this study, together with that obtained with previously described analogs, provide important information for proposing new structures with improved activity.

## Supporting Information

File 1 Experimental procedures, compound characterization data, and copies of NMR spectra.

## Data Availability

All data that supports the findings of this study is available in the published article and/or the supporting information of this article.
