# Supplementary material for: Synthesis of the 1,5-disubstituted tetrazole-methanesulfonylindole hybrid system via high-order multicomponent reaction
Source: Beilstein J Org Chem. 2024 Nov 26;20:3077–84. doi: 10.3762/bjoc.20.256 (PMC11610483; doi:10.3762/bjoc.20.256)
Supplement: File 1 — Experimental procedures, compound characterization data, and copies of NMR spectra. [file Beilstein_J_Org_Chem-20-3077-s001.pdf]

BEILSTEIN JOURNAL OF ORGANIC CHEMISTRY

# Supporting Information

for

# Synthesis of the 1,5-disubstituted tetrazole-methanesulfonylindole hybrid system via high-order multicomponent reaction

Cesia M. Aguilar-Morales, América A. Frías-López, Nadia V. Emilio-Velázquez, Alejandro Islas-Jácome, Angelica Judith Granados-López, Jorge Gustavo Araujo-Huitrado, Yamilé López-Hernández, Hiram Hernández-López, Luis Chacón-García, Jesús Adrián López and Carlos J. Cortés-García

*Beilstein J. Org. Chem.* **2024,** *20,* 3077–3084. doi:10.3762/bjoc.20.256

## Experimental procedures, compound characterization data, and copies of NMR spectra

License and Terms: This is a supporting information file under the terms of the Creative Commons Attribution License (https://creativecommons.org/licenses/by/4.0). Please note that the reuse, redistribution and reproduction in particular requires that the author(s) and source are credited and that individual graphics may be subject to special legal provisions.
The license is subject to the *Beilstein Journal of Organic Chemistry* terms and conditions: (https://www.beilstein-journals.org/bjoc/terms)

## Table of contents

General information ........................................................................................................................ S1

Synthesis of *N*-(2-iodophenyl)methanesulfonamide (**17**) ................................................................................ S2

General procedure for 1,5-disubstituted tetrazol-*N*-mesyl indoles *bis*-heterocycles **18a–n** ............................ S4

Synthesis and NMR spectra of the products **18a–n** ......................................................................................... S4

General information for biological evaluation ............................................................................................. S39

Biological evaluation of compounds **18a–j** ................................................................................................... S39

**General information**

Reagents and solvents were purchased from commercial suppliers and were used without further purification. Reaction progress was monitored by thin-layer chromatography (TLC) using silica gel 60 $F_{254}$ from Merck and the spots were visualized under UV light at 254 or 365 nm. Column chromatography was performed using silica gel (230–400 mesh). Chemical names and drawings were obtained using ChemDraw Professional (version 15.0.0.106). Melting points were determined on a Fisher-Johns melting point apparatus and are uncorrected. HRMS spectra were acquired on a Bruker MicroTOF-II spectrometer. NMR spectra were recorded with a Varian Mercury spectrometer (400 MHz) and a Bruker AMX Advance III spectrometer (500 MHz). Chemical shifts were reported as δ values (ppm). Coupling constants *J* are reported in hertz (Hz). Internal reference for NMR spectra is in respect to TMS at 0.0 ppm. Multiplicities are reported, using the standard abbreviations, as follows: singlet (s), doublet (d), doublet of doublets (dd), triplet of doublet of doublets (tdd), doublet of quartets (dq), doublet of doublet of doublets (ddd), broad signal (bs), multiplet (m), apparent triplet (at). NMR spectra were analyzed using the MestreNova software (version 6.0.2-5475). IR spectra were recorded on a Thermo Scientific NICOLET iS10 by ATR method using neat compounds. The wavelengths are reported in reciprocal centimeters ($\nu$/cm$^{-1}$).

## Synthesis of *N*-(2-iodophenyl)methanesulfonamide (17)

Under a nitrogen atmosphere, $Et_3N$ (95.0 µL, 0.68 mmol) was added at 0–5 °C to a solution of 2-iodoaniline (100 mg, 0.45 mmol) in dichloromethane (0.13 M). Then, sulfonyl chloride (42.0 µL, 0.54 mmol) was added dropwise at 0 °C. After the addition was complete, the reaction mixture was allowed to warm to room temperature until consumption of 2-iodoaniline via TLC. Next, a saturated solution of $NaHCO_3$ (5 mL) was added and extracted with dichloromethane (8 mL). The aqueous phase was extracted twice with dichloromethane (5 mL). The combined organic phases were washed with water (2 × 5 mL), dried over $Na_2SO_4$, filtered, and concentrated under reduced pressure. Finally, the residue was purified by flash column chromatography using a hexane/EtOAc 8:2 mixture (v/v), yielding *N*-(2-iodophenyl)methanesulfonamide (**17**) as a white solid. $R_f$ 0.40 (hexane/AcOEt 7:3, v/v). The $^1H$ and $^{13}C$ NMR spectra were consistent with the data reported by Mondal et al. and Buarque et al. $^1H$ NMR (400 MHz, $CDCl_3$): δ = 7.84 (dd, *J* = 8.3, 1.3 Hz, 1H), 7.66 (dd, *J* = 8.1, 1.1 Hz, 2H), 7.39 (at, *J* = 7.4 Hz, 1H), 6.95 (at, *J* = 7.2 Hz, 1H), 6.65 (bs, 1H), 3.02 (s, 3H). $^{13}C$ NMR (100 MHz, $CDCl_3$): δ = 141.1, 136.9, 131.8, 131.5, 129.4, 103.1, 43.8.

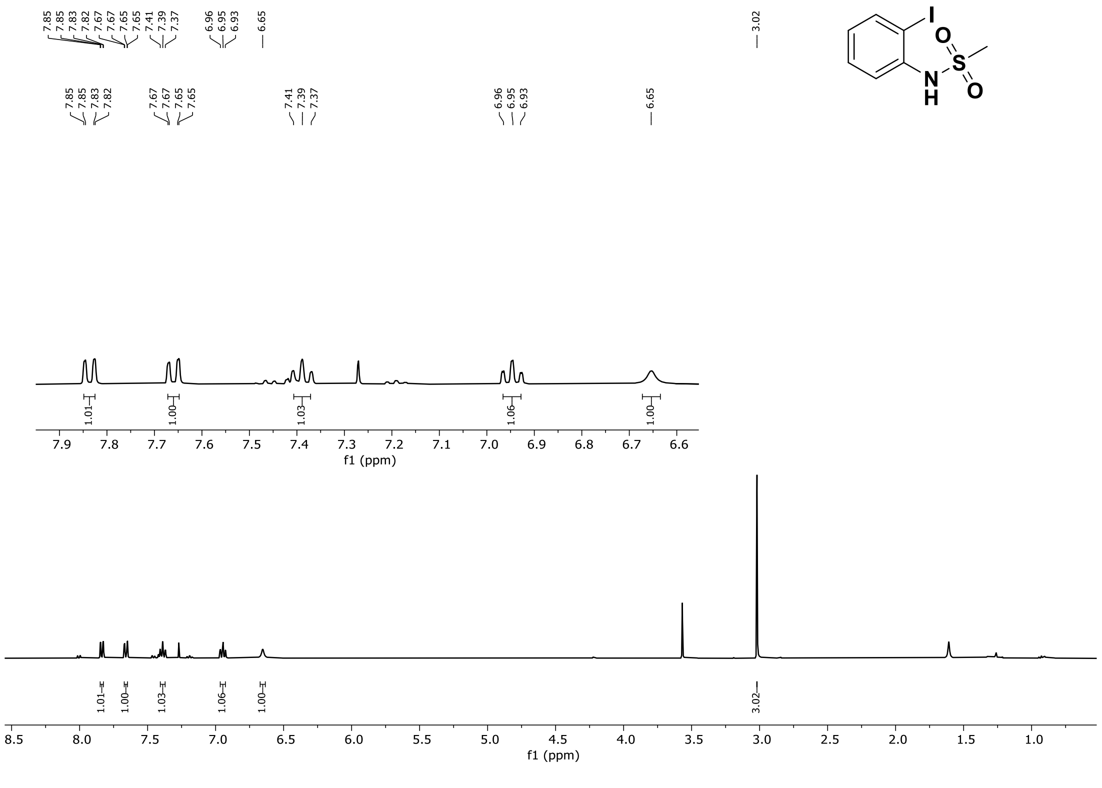


$^1H$ NMR spectra of the compound **17**

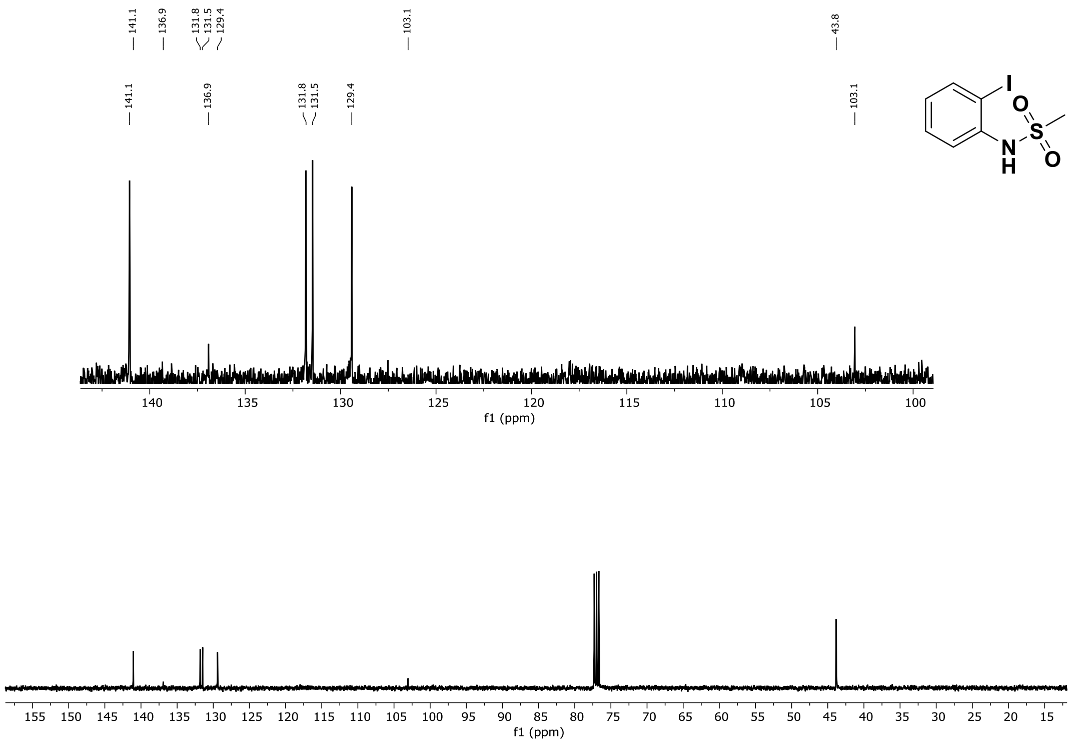


$^{13}$C NMR spectra of the compound **17**

**General procedure for 1,5-disubstituted tetrazol-*N*-mesyl indoles *bis*-heterocycles 18a–n (GP)**

**Note:** The general procedure reported herein follows a method analogous to that described recently by the Cortés-García research group.[3]

In a dry pressure tube, propargylamine (1.0 equiv) and aldehyde (1.0 equiv) were dissolved on TFE (1 M) and reacted for 5 min at room temperature. Sequentially, isocyanide (1.0 equiv) and $TMSN_3$ (1.2 equiv) were added and stirred at room temperature until complete by TLC (approximately 24 h). Later, the reaction mixture was evaporated under reduced pressure. The residue was suspended on dry DMF (0.8 M) and $(PPh_3)_2PdCl_2$ (0.1 equiv), CuI (0.08 equiv), $Et_3N$ (0.1 M), and *N*-(2-iodophenyl)methanesulfonamide (**17**, 1.2 equiv) were loaded, and deoxygenated via cannula under a nitrogen atmosphere. Next, the reaction mixture was heated to 70 °C for 1 hour. Finally, ethyl acetate (3 mL) was added and filtration through a pad of celite and silica gel was done. The liquor mothers were poured into a mixture of water (7 mL) and ethyl acetate (5 mL). The aqueous phase was extracted twice with ethyl acetate (5 mL). The combined organic phases were washed with water (2 × 5 mL), dried over $Na_2SO_4$, filtered, and concentrated under reduced pressure. Purification by flash column chromatography (hexane/EtOAc 7:3, v/v) afforded the 1,5-disubstituted tetrazol-*N*-mesyl indoles **18a**–**n**.

1-(1-(*tert*-Butyl)-1*H*-tetrazol-5-yl)-1-(4-fluorophenyl)-*N*-((1-(methylsulfonyl)-1*H*-indol-2-yl)methyl)methanamine (**18a)**

Based on the GP, propargylamine (23.3 µL, 0.36 mmol), 4-fluorobenzaldehyde (39.0 µL, 0.36 mmol), $TMSN_3$ (58.0 µL, 0.43 mmol), *tert*-butyl isocyanide (41.0 µL, 0.36 mmol), *N*-(2-iodophenyl)methanesulfonamide (129.4 mg, 0.43 mmol), $(PPh_3)_2PdCl_2$ (25.5 mg, 0.04 mmol) and CuI (5.5 mg, 0.03 mmol) were used and **18a** was obtained as a yellow semisolid (53.0 mg, 32%). $R_f$ = 0.46 (Hexane-AcOEt 7:3 v/v); $^1$H NMR (400 MHz, $CDCl_3$): δ = 8.00 (d, *J* = 8.3 Hz, 1H), 7.50 (d, *J* = 7.6 Hz, 1H), 7.33–7.30 (m, 3H), 7.27–7.25 (m, 1H), 7.04 (at, *J* = 8.5 Hz, 2H), 6.50 (s, 1H), 5.52 (s, 1H), 4.10 (d, *J* = 13.7 Hz, 1H), 3.97 (d, *J* = 13.7 Hz, 1H), 3.21 (s, 3H), 1.63 (s, 9H). $^{13}$C NMR (100 MHz, $CDCl_3$): δ = 162.5 (d, *J* = 248.4 Hz), 154.9, 138.3, 136.9, 134.4 (d, *J* = 3.4 Hz), 129.8 (d, *J* = 8.2 Hz), 128.5, 124.7, 123.5, 120.9, 116.1 (d, *J* = 21.7 Hz), 113.8, 110.1, 61.5, 58.3, 45.0, 40.9, 29.9. FT-IR (ATR) $\nu_{max}/cm^{-1}$ 3302, 2917, 1603, 1509, 1350, 1221, 1053. HRMS ($ESI^+$): m/z: Calcd. for $C_{22}H_{26}FN_6O_2S$ $[M+H]^+$: 457.1816; Found: 457.1823.

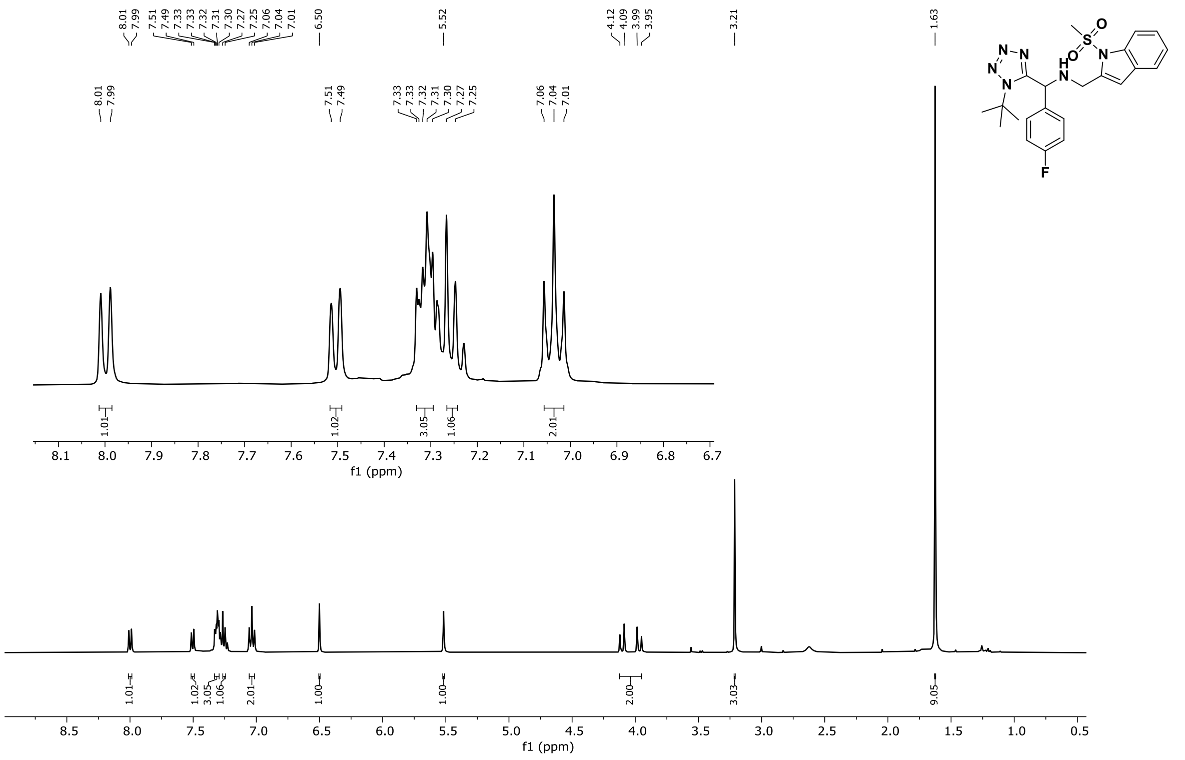


$^{1}$H NMR spectra of the compound **18a**

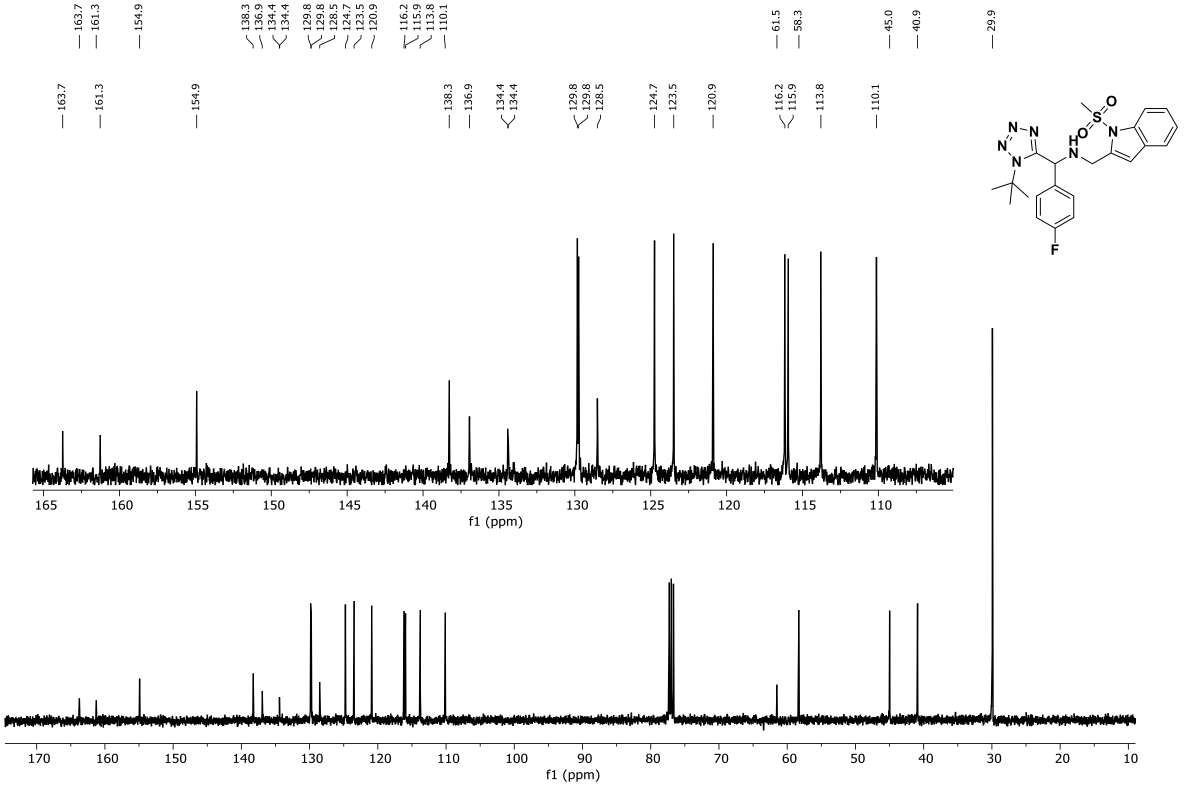


$^{13}$C NMR spectra of the compound **18a**

1-(1-Cyclohexyl-1*H*-tetrazol-5-yl)-1-(4-fluorophenyl)-*N*-((1-(methylsulfonyl)-1*H*-indol-2-yl)methyl)methanamine (**18b)**

Based on the GP, propargylamine (23.3 µL, 0.36 mmol), 4-fluorobenzaldehyde (39.0 µL, 0.36 mmol), $TMSN_3$ (58.0 µL, 0.43 mmol), cyclohexyl isocyanide (45.1 µL, 0.36 mmol), *N*-(2-iodophenyl)methanesulfonamide (129.4 mg, 0.43 mmol), $(PPh_3)_2PdCl_2$ (25.5 mg, 0.04 mmol) and CuI (5.5 mg, 0.03 mmol) were used and **18b** was obtained as an ambar oil (52.5 mg, 30%). $R_f$ = 0.43 (Hexane-AcOEt 7:3 v/v); $^1$H NMR (400 MHz, $CDCl_3$): δ = 7.99 (d, *J* = 8.3 Hz, 1H), 7.52 (d, *J* = 7.0 Hz, 1H), 7.32–7.29 (m, 3H), 7.27–7.25 (m, 1H), 7.05 (at, *J* = 8.6 Hz, 2H), 6.52 (s, 1H), 5.26 (s, 1H), 4.17 (d, *J* = 14.5 Hz, 1H), 4.11–4.08 (m, 1H), 3.98 (d, *J* = 14.3 Hz, 1H), 3.16 (s, 3H), 1.93–1.18 (m, 10H). $^{13}$C NMR (100 MHz, $CDCl_3$): δ = 162.9 (d, *J* = 248.6 Hz), 154.4, 138.0, 137.3, 134.0 (d, *J* = 2.8 Hz), 129.4 (d, *J* = 8.3 Hz), 128.9, 125.2, 124.0, 121.3, 116.4 (d, *J* = 21.7 Hz), 114.2, 111.4, 58.3, 56.1, 44.8, 41.1, 32.8, 25.4, 24.9. FT-IR (ATR) $\nu_{max}$/cm$^{-1}$ 3320, 2932, 2854, 1608, 1511, 1458, 1362, 1165, 1054. HRMS (ESI$^+$): m/z: Calcd. for $C_{24}H_{28}FN_6O_2S$ [M+H]$^+$: 483.1973; Found: 483.1979.

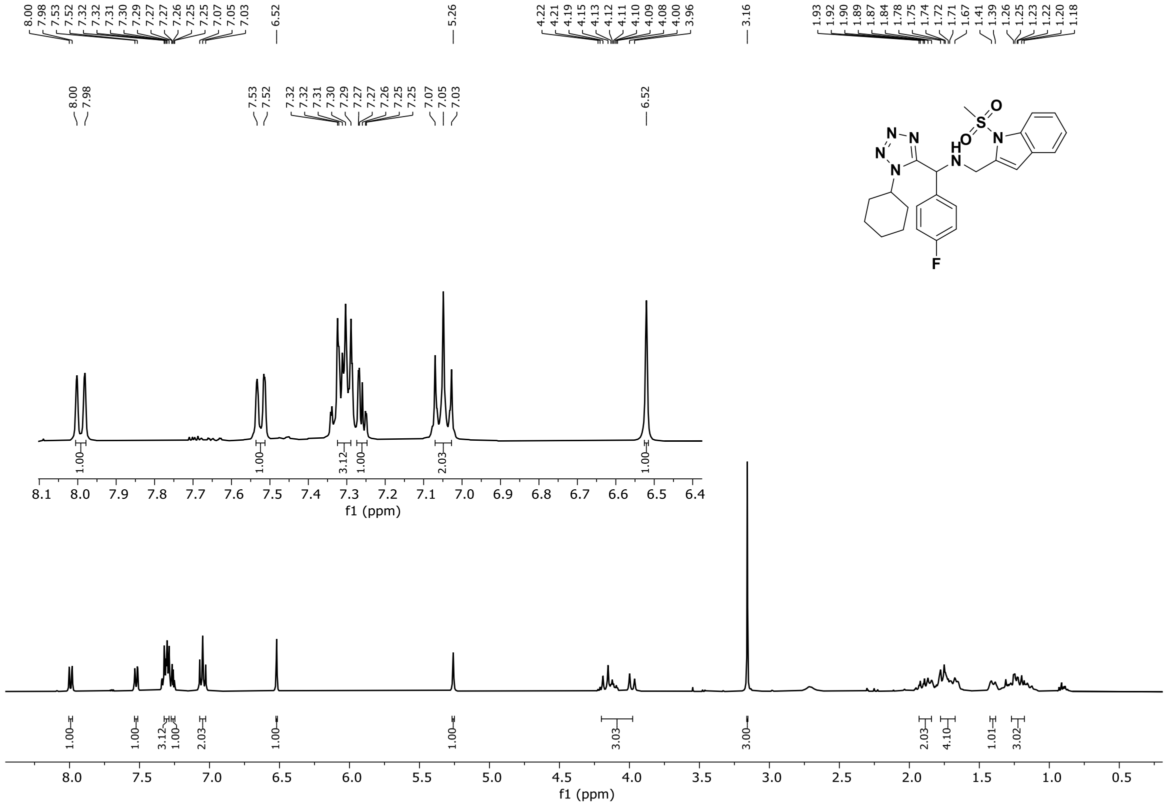


$^1$H NMR spectra of the compound **18b**

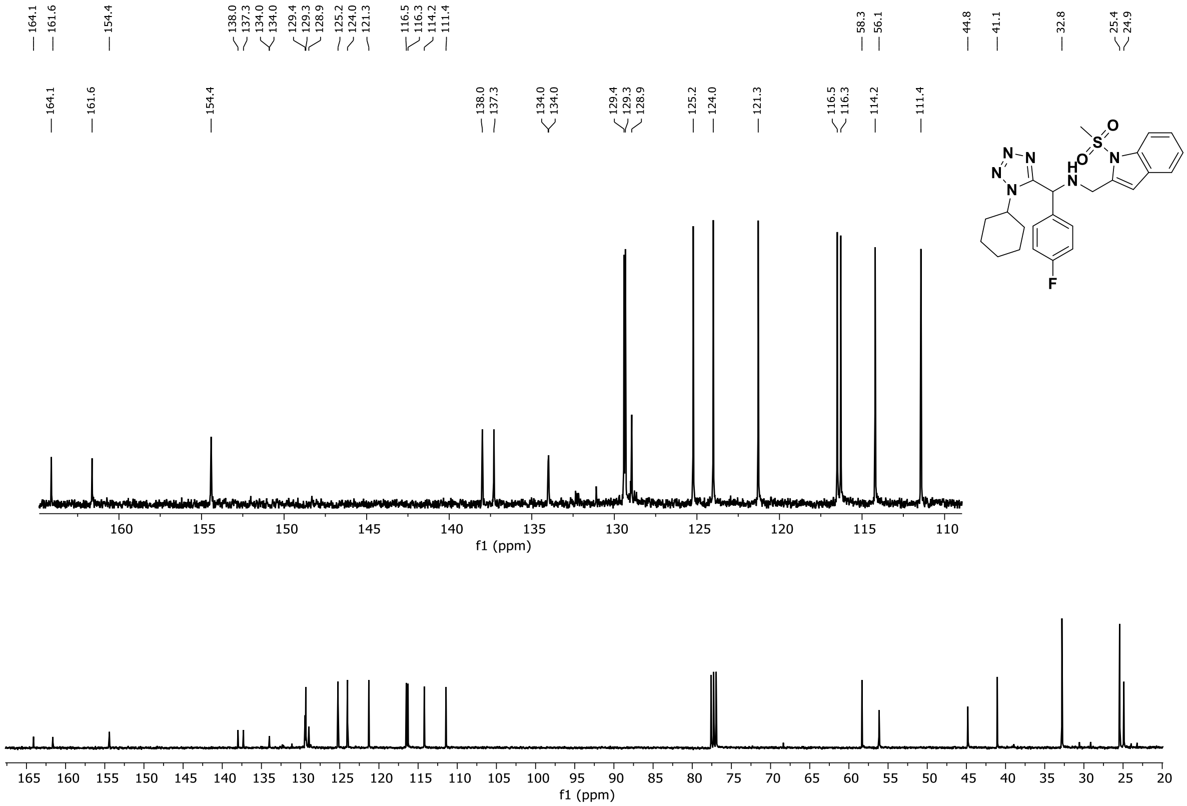

$^{13}$C NMR spectra of the compound **18b**

1-(1-(*tert*-Butyl)-1*H*-tetrazol-5-yl)-1-(2-fluorophenyl)-*N*-((1-(methylsulfonyl)-1*H*-indol-2-yl)methyl)methanamine (**18c)**

Based on the GP, propargylamine (23.3 µL, 0.36 mmol), 2-fluorobenzaldehyde (38.2 µL, 0.36 mmol), $TMSN_3$ (58.0 µL, 0.43 mmol), *tert*-butyl isocyanide (41.0 µL, 0.36 mmol), *N*-(2-iodophenyl)methanesulfonamide (129.4 mg, 0.43 mmol), $(PPh_3)_2PdCl_2$ (25.5 mg, 0.04 mmol) and CuI (5.5 mg, 0.03 mmol) were used and **18c** was obtained as a beige solid (87.8 mg, 53%). mp = 143–145 °C. $R_f$ = 0.46 (Hexane-AcOEt 7:3 v/v); $^1$H NMR (400 MHz, $CDCl_3$): δ = 8.00 (d, *J* = 8.4 Hz, 1H), 7.50 (d, *J* = 8.1 Hz, 1H), 7.34–7.28 (m, 3H), 7.26–7.22 (m, 1H), 7.14–7.09 (m, 2H), 6.51 (s, 1H), 5.95 (s, 1H), 4.17 (d, *J* = 13.8 Hz, 1H), 4.02 (d, *J* = 13.9 Hz, 1H), 3.24 (s, 3H), 1.62 (s, 9H). $^{13}$C NMR (100 MHz, $CDCl_3$): δ = 159.5 (d, *J* = 246.9 Hz), 154.4, 138.3, 137.0, 130.4 (d, *J* = 8.4 Hz), 129.0 (d, *J* = 3.0 Hz), 128.5, 125.7 (d, *J* = 14.4 Hz), 125.2 (d, *J* = 3.6 Hz), 124.7, 123.4, 120.8, 115.7 (d, *J* = 22.2 Hz), 113.8, 110.0, 61.6, 50.4, 44.6, 40.9, 29.7. FT-IR (ATR) $\nu_{max}$/cm$^{-1}$ 3306, 2975, 2921, 1499, 1358, 1231, 1176, 1057. HRMS ($ESI^+$): m/z: Calcd. for $C_{22}H_{26}FN_6O_2S[M+H]^+$: 457.1816; Found: 457.1801.

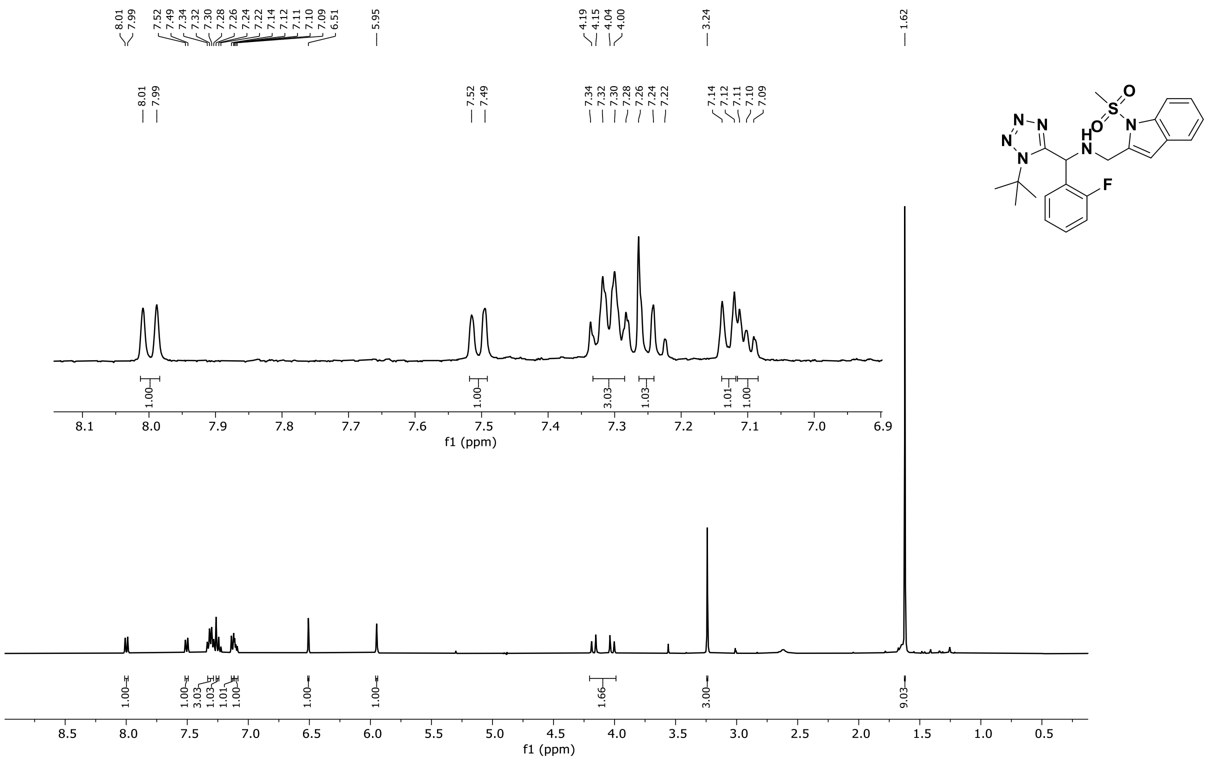

$^1$H NMR spectra of the compound **18c**

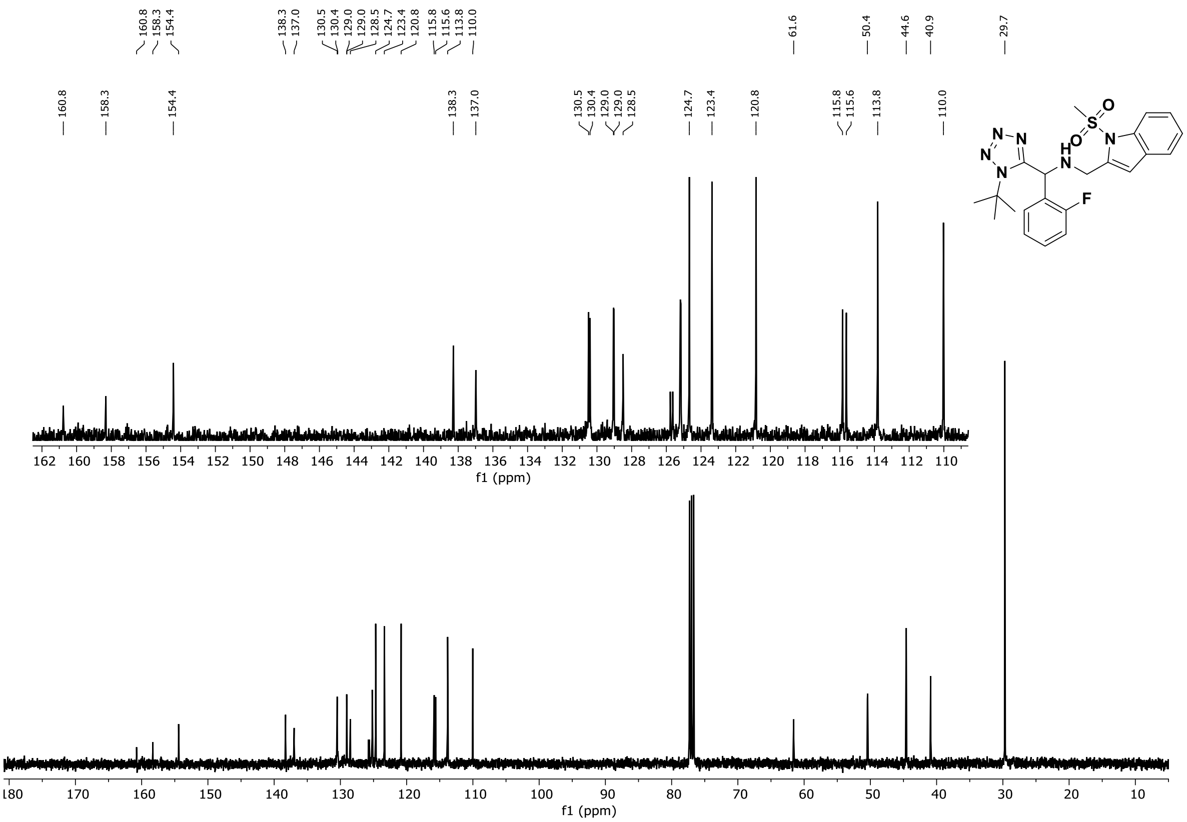

$^{13}$C NMR spectra of the compound **18c**

1-(1-Cyclohexyl-1*H*-tetrazol-5-yl)-1-(2-fluorophenyl)-*N*-((1-(methylsulfonyl)-1*H*-indol-2-yl)methyl)methanamine (**18d)**

Based on the GP, propargylamine (23.3 µL, 0.36 mmol), 2-fluorobenzaldehyde (38.2 µL, 0.36 mmol), $TMSN_3$ (58.0 µL, 0.43 mmol), cyclohexyl isocyanide (45.1 µL, 0.36 mmol), *N*-(2-iodophenyl)methanesulfonamide (129.4 mg, 0.43 mmol), $(PPh_3)_2PdCl_2$ (25.5 mg, 0.04 mmol) and CuI (5.5 mg, 0.03 mmol) were used and **18d** was obtained as a yellow semisolid (54.3 mg, 31%). $R_f$ = 0.36 (Hexane-AcOEt 7:3 v/v); $^1$H NMR (400 MHz, $CDCl_3$): δ = 8.00 (d, *J* = 8.2 Hz, 1H), 7.53 (d, *J* = 7.6 Hz, 1H), 7.39–7.35 (m, 1H), 7.34–7.30 (m, 2H), 7.28–7.25 (m, 2H), 7.17 (at, *J* = 7.5 Hz, 1H), 7.10 (at, *J* = 9.2 Hz, 1H), 6.54 (s, 1H), 5.59 (s, 1H), 4.19–4.05 (m, 3H), 3.21 (s, 3H), 2.78 (bs, 1H), 1.93–1.12 (m, 10H). $^{13}$C NMR (100 MHz, $CDCl_3$): δ = 160.0 (d, *J* = 246.8 Hz), 154.0, 137.9, 137.0, 130.5 (d, *J* = 8.3 Hz), 128.8 (d, *J* = 3.0 Hz), 128.6, 125.2 (d, *J* = 3.5 Hz), 125.0 (d, *J* = 13.6 Hz), 124.8, 123.6, 120.9, 115.7 (d, *J* = 21.9 Hz), 113.9, 110.7, 58.0, 49.0, 49.0, 44.4, 40.9, 32.6, 32.5, 25.2, 25.1, 24.7. FT-IR (ATR) $\nu_{max}$/cm$^{-1}$ 3308, 2919, 2854, 1728, 1589, 1452, 1364, 1174, 1059. HRMS (ESI$^+$): m/z: Calcd. for $C_{24}H_{28}FN_6O_2S[M+H]^+$: 483.1973; Found: 483.1986.

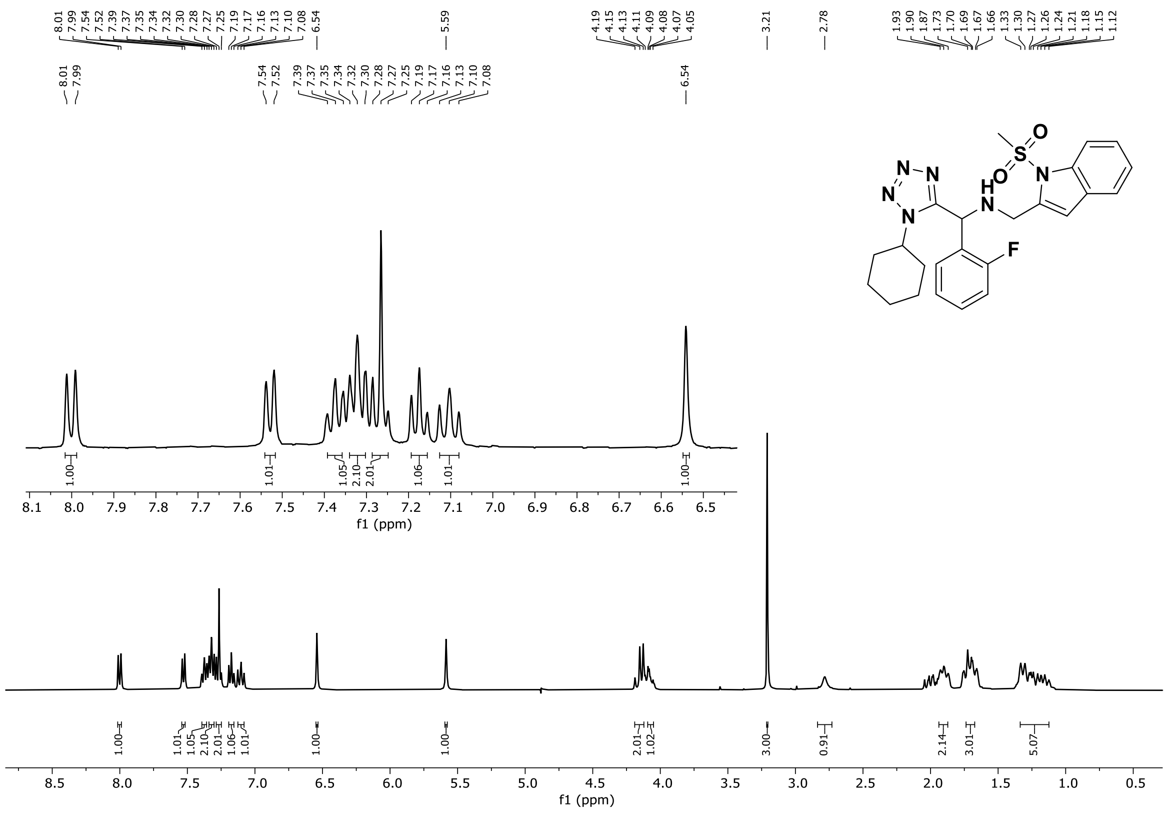


$^1$H NMR spectra of the compound **18d**

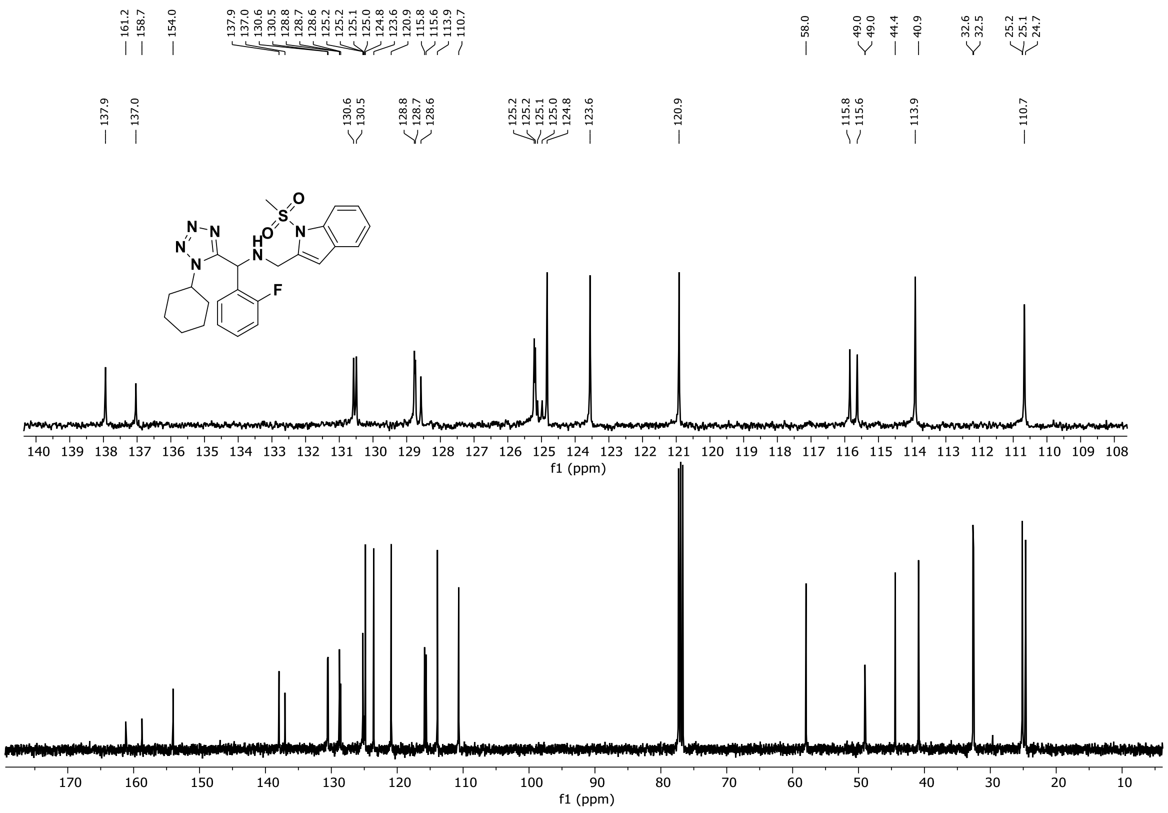

$^{13}C$ NMR spectra of the compound **18d**

1-(1-(*tert*-Butyl)-1*H*-tetrazol-5-yl)-1-(3-fluorophenyl)-*N*-((1-(methylsulfonyl)-1*H*-indol-2-yl)methyl)methanamine (**18e**)

Based on the GP, propargylamine (23.3 µL, 0.36 mmol), 3-fluorobenzaldehyde (38.4 µL, 0.36 mmol), $TMSN_3$ (58.0 µL, 0.43 mmol), *tert*-butyl isocyanide (41.0 µL, 0.36 mmol), *N*-(2-iodophenyl)methanesulfonamide (129.4 mg, 0.43 mmol), $(PPh_3)_2PdCl_2$ (25.5 mg, 0.04 mmol) and CuI (5.5 mg, 0.03 mmol) were used and **18e** was obtained as a yellow solid (54.6 mg, 33%). mp = 146–148 °C. $R_f$ = 0.43 (Hexane-AcOEt 7:3 v/v); $^{1}$H NMR (400 MHz, $CDCl_3$): δ = 8.00 (dq, *J* = 8.4, 0.9 Hz, 1H), 7.51 (ddd, *J* = 7.7, 1.4, 0.7 Hz, 1H), 7.35–7.29 (m, 2H), 7.26–7.23 (m, 1H), 7.10–7.08 (m, 2H), 7.01 (tdd, *J* = 8.4, 2.2, 1.4 Hz, 1H), 6.50 (d, *J* = 0.8 Hz, 1H), 5.52 (s, 1H), 4.11 (d, *J* = 13.7 Hz, 1H), 3.98 (d, *J* = 13.6 Hz, 1H), 3.21 (s, 3H), 2.65 (bs, 1H), 1.64 (s, 9H). $^{13}$C NMR (100 MHz, $CDCl_3$): δ = 163.0 (d, *J* = 248.1 Hz), 154.6, 141.0 (d, *J* = 6.7 Hz), 138.2, 136.9, 130.6 (d, *J* = 8.2 Hz), 128.5, 124.7, 123.6 (d, *J* = 2.9 Hz), 123.5, 120.9, 115.6 (d, *J* = 21.2 Hz), 115.1 (d, *J* = 22.3 Hz), 113.8, 110.1, 61.5, 58.5, 44.9, 40.8, 29.9. FT-IR (ATR) $\nu_{max}$/$cm^{-1}$ 3300, 2964, 2930, 1587, 1458, 1356, 1155, 1055. HRMS ($ESI^+$): m/z: Calcd. for $C_{22}H_{26}FN_6O_2S[M+H]^+$: 457.1816; Found: 457.1838.

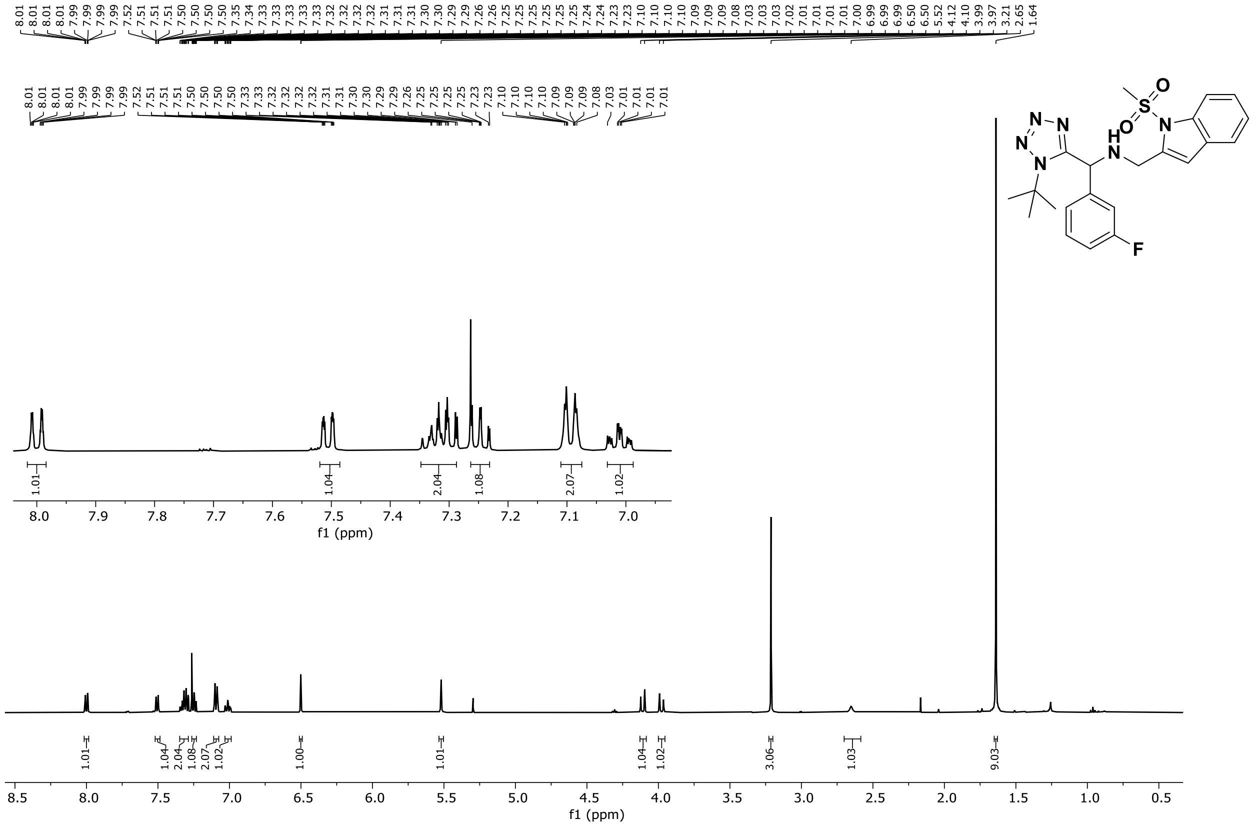


$^{1}$H NMR spectra of the compound **18e**

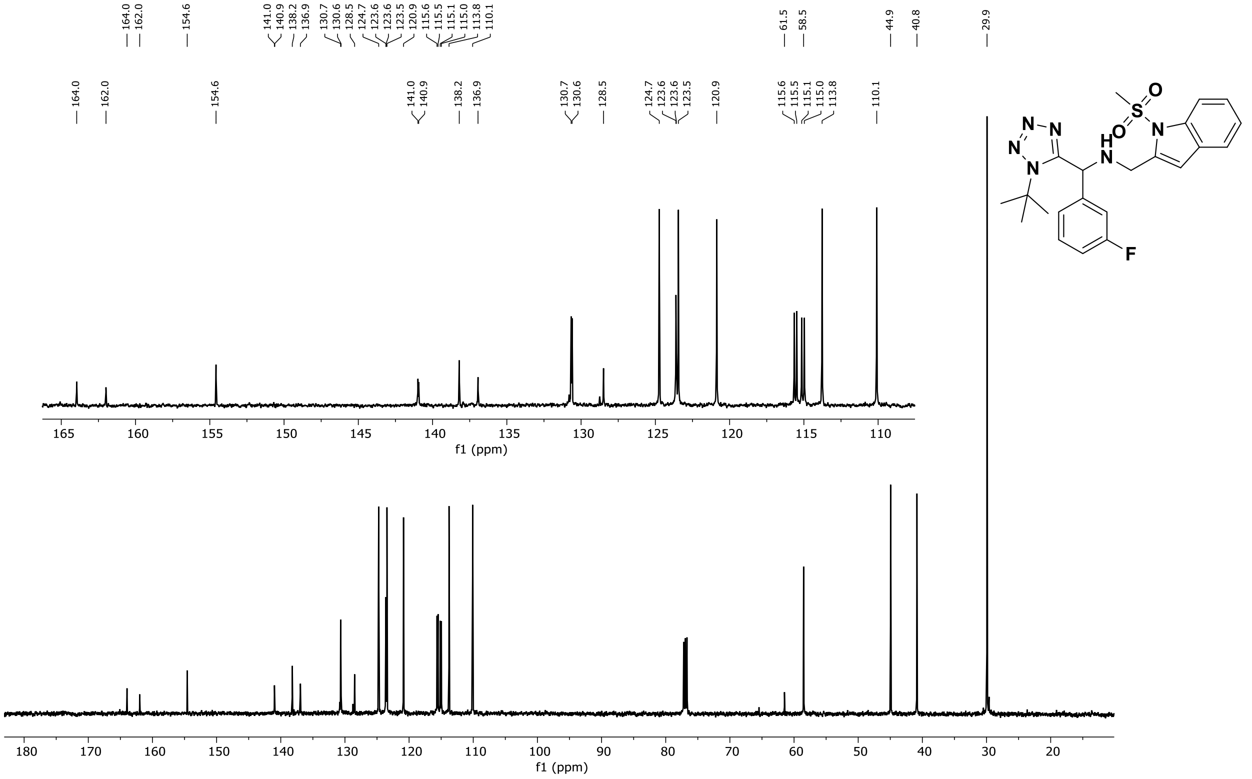


$^{13}$C NMR spectra of the compound **18e**

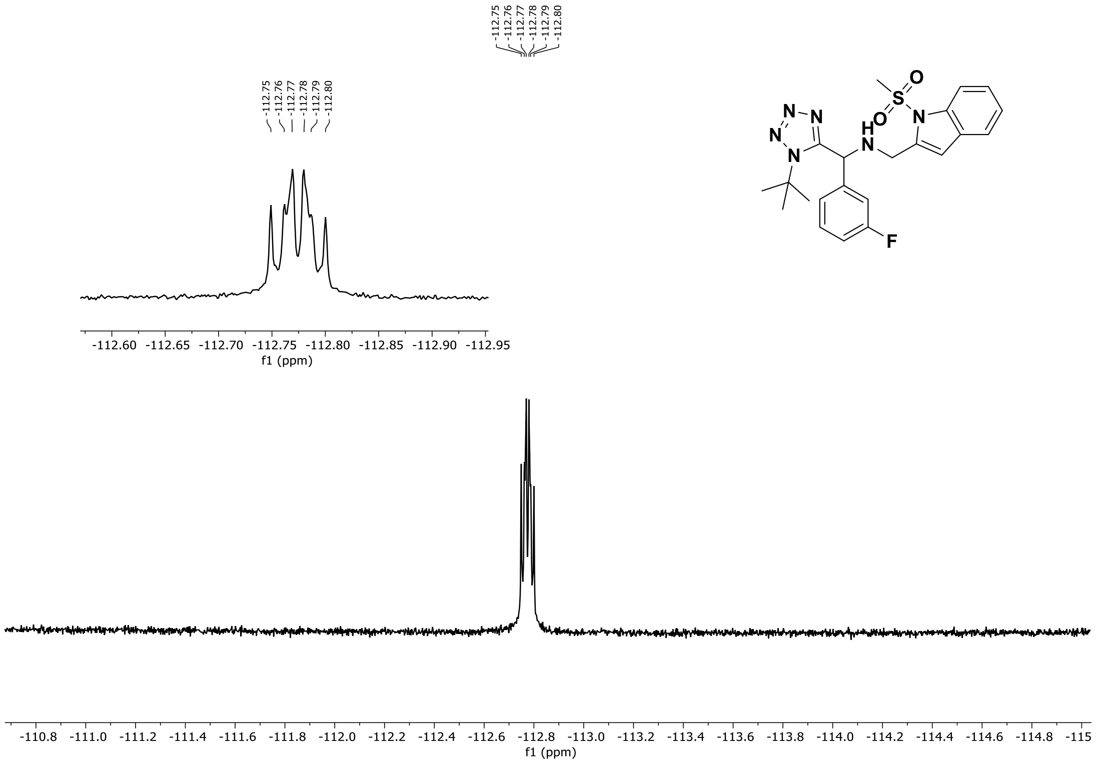


$^{19}$F NMR spectra of the compound **18e**

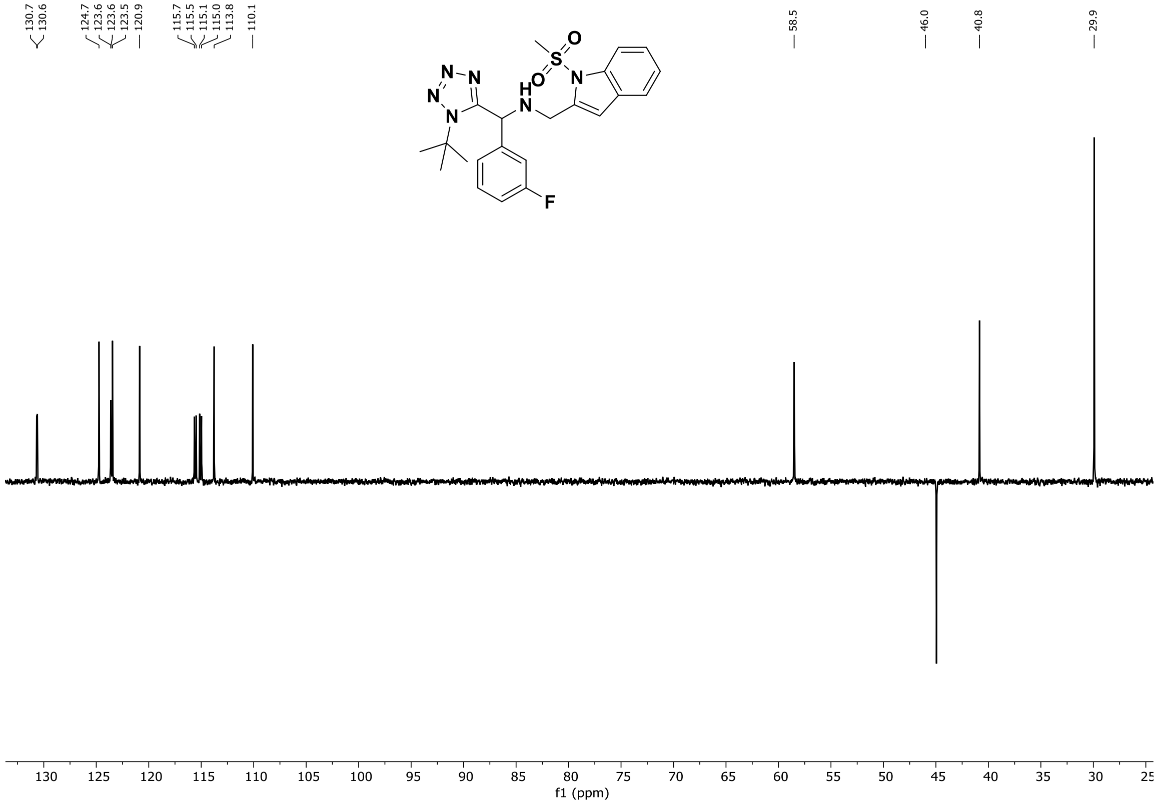


DEPT-135 NMR spectra of the compound **18e**

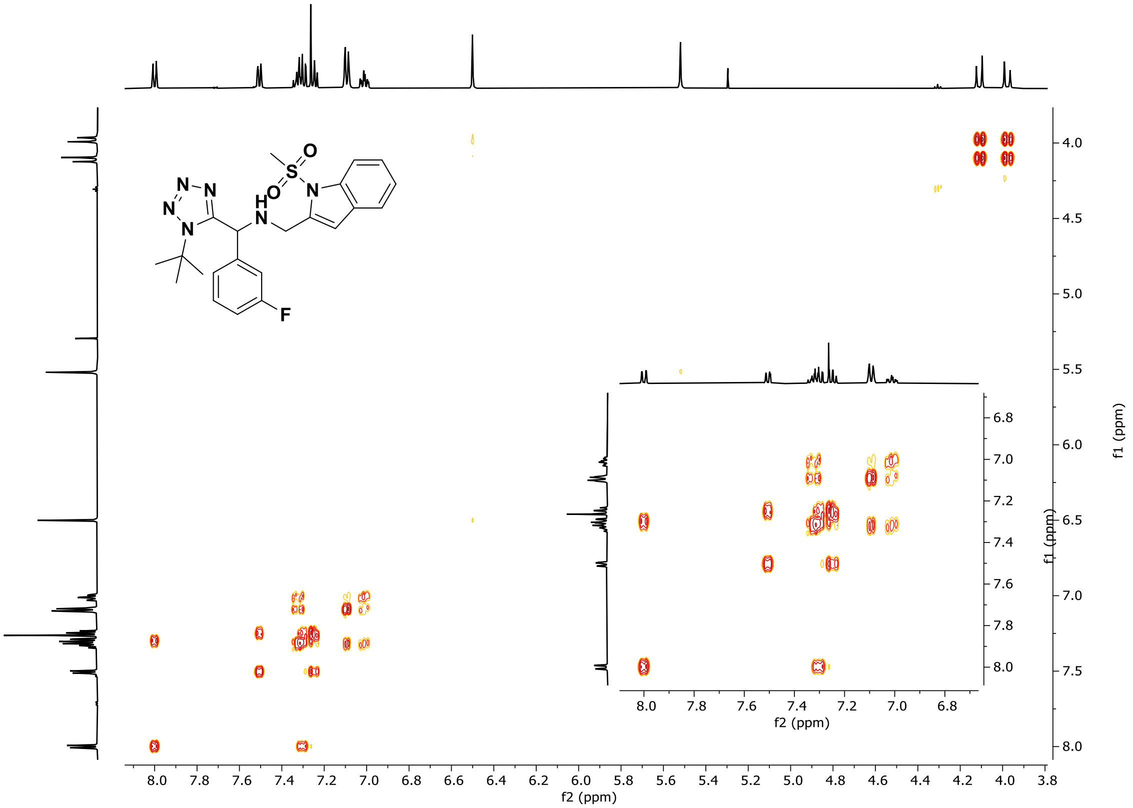


COSY NMR spectra of the compound **18e**

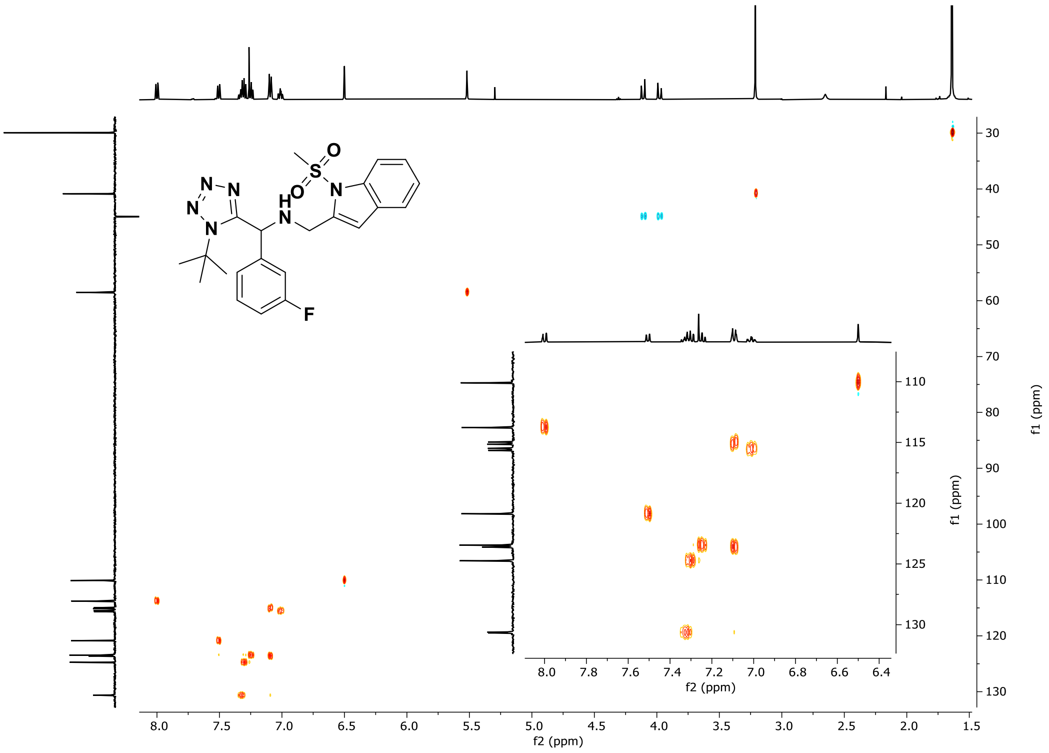

HSQC NMR spectra of the compound **18e**

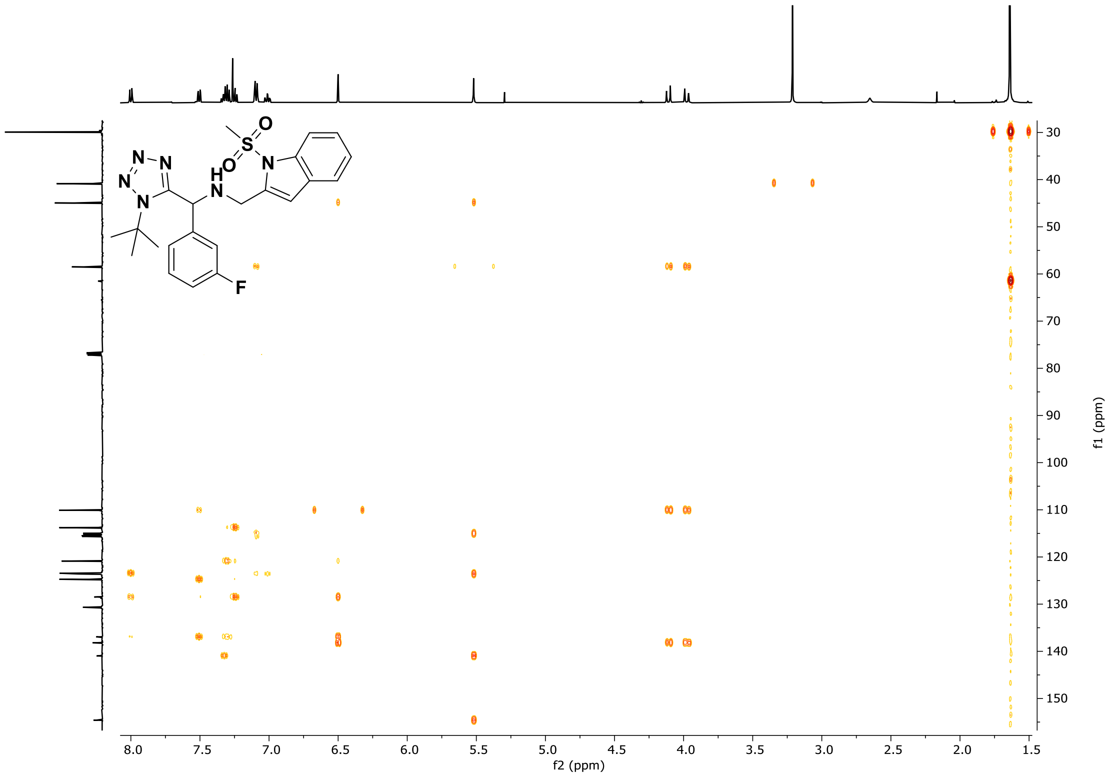

HMBC NMR spectra of the compound **18e**

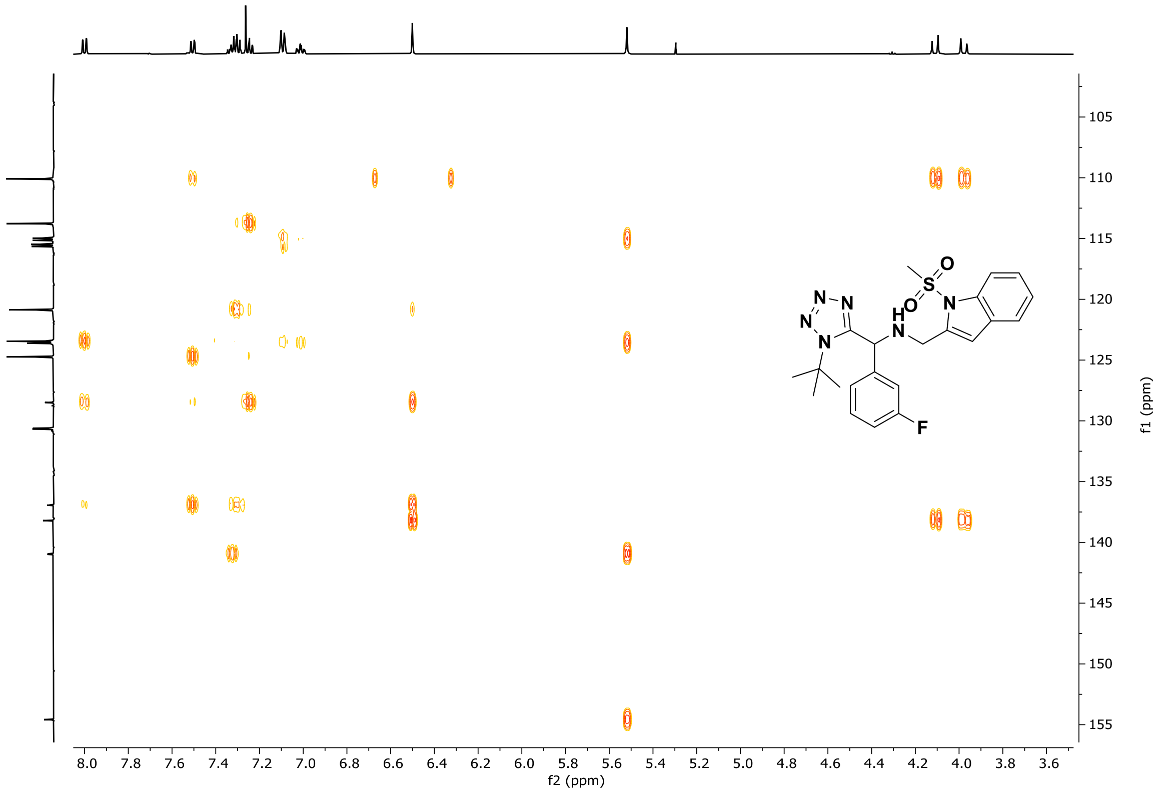


HMBC NMR spectra of the compound **18e** (expansion)

1-(1-Cyclohexyl-1*H*-tetrazol-5-yl)-1-(3-fluorophenyl)-*N*-((1-(methylsulfonyl)-1*H*-indol-2-yl)methyl)methanamine (**18f)**

Based on the GP, propargylamine (23.3 µL, 0.36 mmol), 3-fluorobenzaldehyde (38.4 µL, 0.36 mmol), $TMSN_3$ (58.0 µL, 0.43 mmol), cyclohexyl isocyanide (45.1 µL, 0.36 mmol), *N*-(2-iodophenyl)methanesulfonamide (129.4 mg, 0.43 mmol), $(PPh_3)_2PdCl_2$ (25.5 mg, 0.04 mmol) and CuI (5.5 mg, 0.03 mmol) were used and **18f** was obtained as a yellow solid (59.5 mg, 34%). mp = 133–135 °C. $R_f$ = 0.40 (Hexane-AcOEt 7:3 v/v); $^1H$ NMR (400 MHz, $CDCl_3$): δ = 7.99 (d, *J* = 8.2 Hz, 1H), 7.53 (d, *J* = 7.2 Hz, 1H), 7.36–7.32 (m, 1H), 7.30–7.25 (m, 3H), 7.10–7.07 (m, 2H), 7.04–6.99 (m, 1H), 6.54 (s, 1H), 5.29 (s, 1H), 4.18 (d, *J* = 13.8 Hz, 2H), 4.16–4.12 (m, 1H), 3.99 (d, *J* = 14.3 Hz, 1H), 3.15 (s, 3H), 2.74 (bs, 1H), 1.91–1.18 (m, 10H). $^{13}C$ NMR (100 MHz, $CDCl_3$): δ = 163.3 (d, *J* = 248.5 Hz), 154.1, 140.6 (d, *J* = 6.7 Hz), 137.8, 137.3, 131.0 (d, *J* = 8.2 Hz), 128.9, 125.2, 124.0, 123.2 (d, *J* = 2.9 Hz), 121.3, 116.0 (d, *J* = 21.1 Hz), 114.6 (d, *J* = 22.4 Hz), 114.2, 111.5, 58.4, 56.3, 44.8, 41.0, 32.8, 25.4, 24.9. FT-IR (ATR) $\nu_{max}/cm^{-1}$ 3341, 2932, 2856, 1589, 1450, 1358, 1170, 1061. HRMS ($ESI^+$): m/z: Calcd. for $C_{24}H_{28}FN_6O_2S[M+H]^+$: 483.1973; Found: 483.1970.

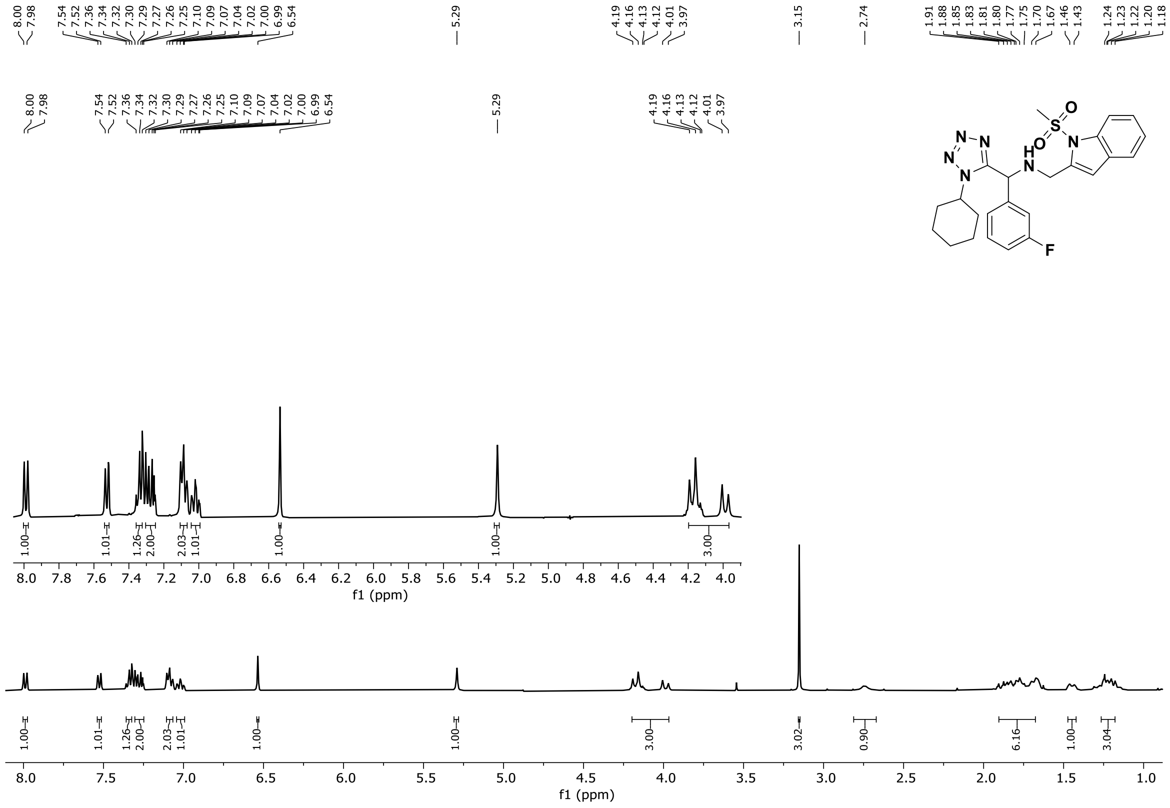


$^1H$ NMR spectra of the compound **18f**

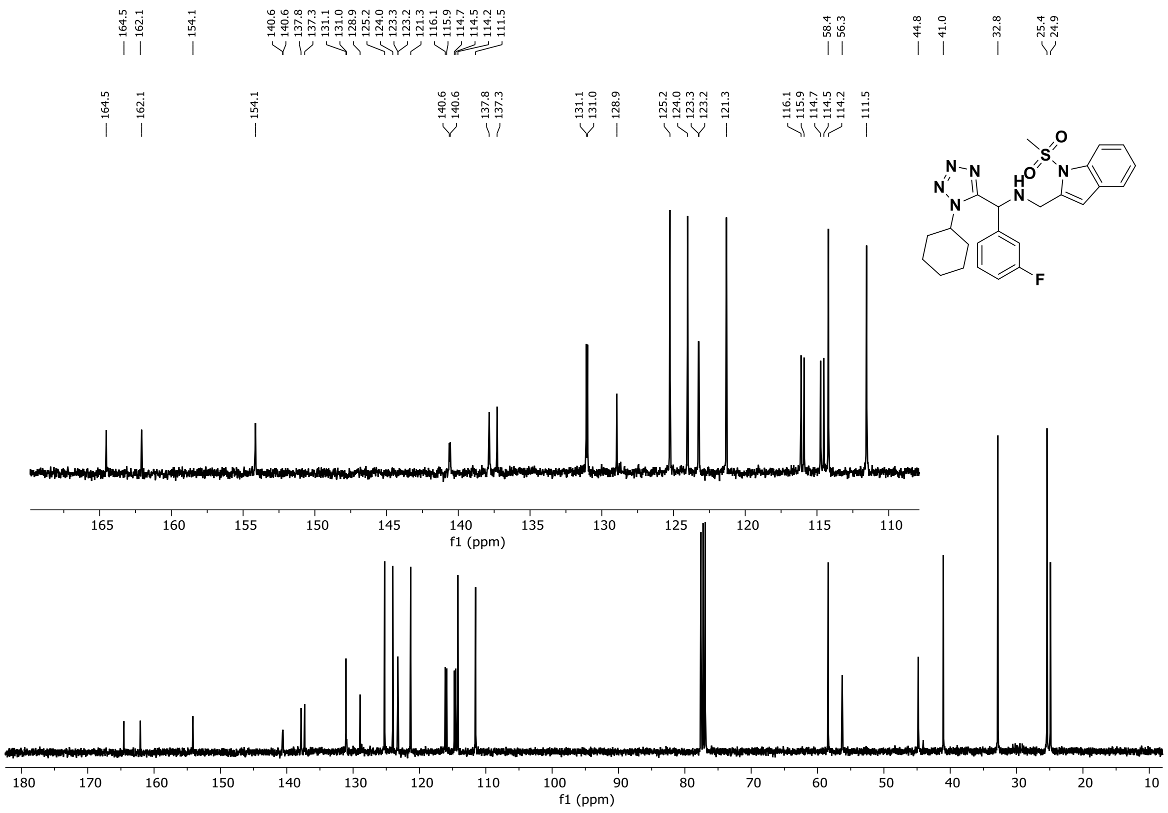

$^{13}C$ NMR spectra of the compound **18f**

1-(1-(*tert*-Butyl)-1*H*-tetrazol-5-yl)-1-(3-chlorophenyl)-*N*-((1-(methylsulfonyl)-1*H*-indol-2-yl)methyl)methanamine (**18g)**

Based on the GP, propargylamine (23.3 µL, 0.36 mmol), 3-chlorobenzaldehyde (41.0 µL, 0.36 mmol), $TMSN_3$ (58.0 µL, 0.43 mmol), *tert*-butyl isocyanide (41.0 µL, 0.36 mmol), *N*-(2-iodophenyl)methanesulfonamide (129.4 mg, 0.43 mmol), $(PPh_3)_2PdCl_2$ (25.5 mg, 0.04 mmol) and CuI (5.5 mg, 0.03 mmol) were used and **18g** was obtained as a yellow solid (94.4 mg, 55%). mp = 82–85 °C. $R_f$ = 0.50 (Hexane-AcOEt 7:3 v/v); $^1$H NMR (400 MHz, $CDCl_3$): δ = 7.99 (d, *J* = 8.4 Hz, 1H), 7.50 (d, *J* = 7.7 Hz, 1H), 7.38 (s, 1H), 7.32–7.26 (m, 3H), 7.24–7.22 (m, 1H), 7.18–7.16 (m, 1H), 6.49 (s, 1H), 5.50 (s, 1H), 4.09 (d, *J* = 13.6 Hz, 1H), 3.97 (d, *J* = 13.6 Hz, 1H), 3.21 (s, 3H), 1.63 (s, 9H). $^{13}$C NMR (100 MHz, $CDCl_3$): δ = 154.8, 140.8, 138.4, 137.2, 135.3, 130.6, 129.0, 128.8, 128.5, 126.3, 125.1, 123.8, 121.2, 114.1, 110.4, 61.8, 58.8, 45.3, 41.1, 30.3. FT-IR (ATR) $\nu_{max}$/cm$^{-1}$ 3306, 2993, 1599, 1575, 1458, 1356, 1170, 1057. HRMS (ESI$^+$): m/z: Calcd. for $C_{22}H_{26}ClN_6O_2S$ [M+H]$^+$: 473.1521; Found: 473.1541.

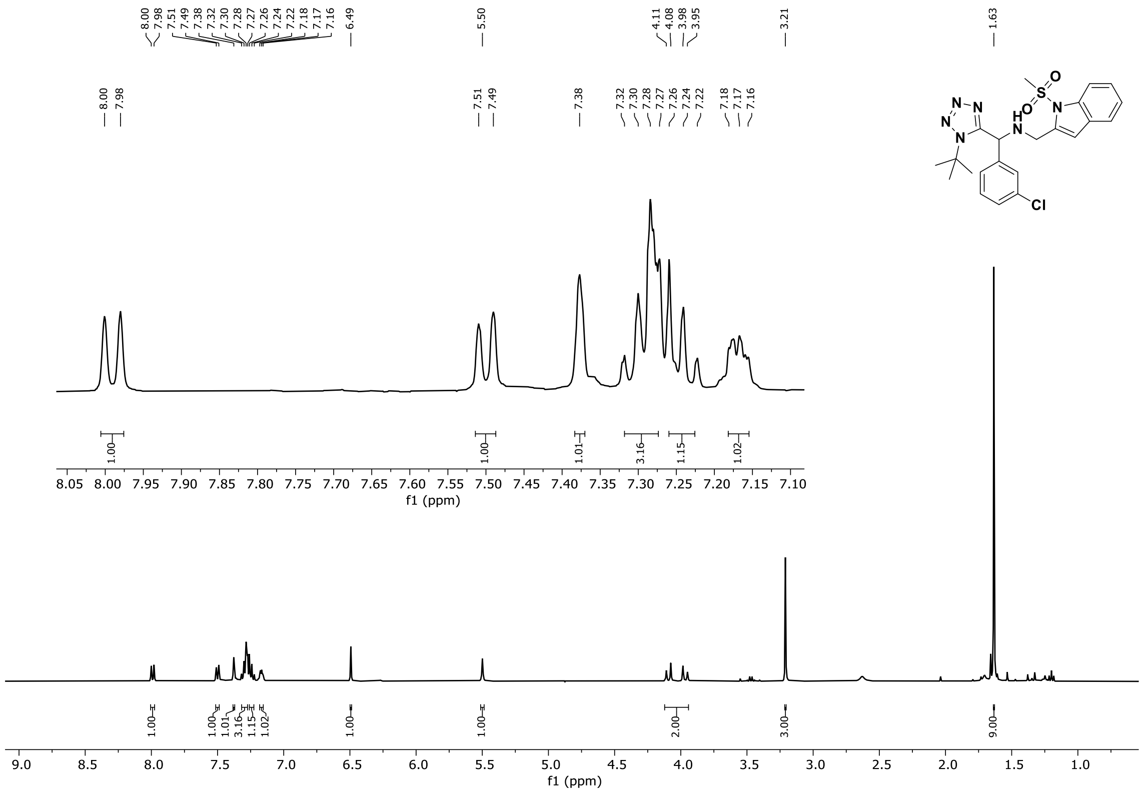


$^1$H NMR spectra of the compound **18g**

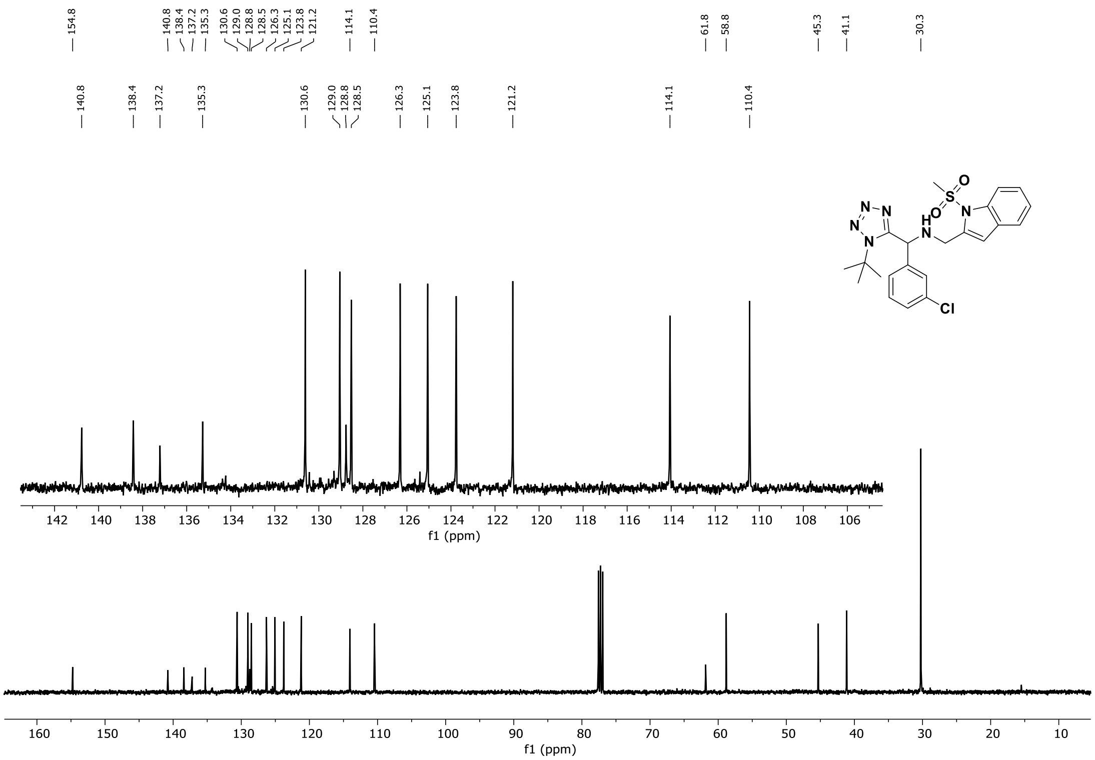


$^{13}C$ NMR spectra of the compound **18g**

1-(3-Chlorophenyl)-1-(1-cyclohexyl-1*H*-tetrazol-5-yl)-*N*-((1-(methylsulfonyl)-1*H*-indol-2-yl)methyl)methanamine (**18h)**

Based on the GP, propargylamine (23.3 µL, 0.36 mmol), 3-chlorobenzaldehyde (41.0 µL, 0.36 mmol), $TMSN_3$ (58.0 µL, 0.43 mmol), cyclohexyl isocyanide (45.1 µL, 0.36 mmol), *N*-(2-iodophenyl)methanesulfonamide (129.4 mg, 0.43 mmol), $(PPh_3)_2PdCl_2$ (25.5 mg, 0.04 mmol) and CuI (5.5 mg, 0.03 mmol) were used and **18h** was obtained as a yellow solid (59.7 mg, 33%). mp = 68–70 °C. $R_f$ = 0.46 (Hexane-AcOEt 7:3 v/v); $^1$H NMR (400 MHz, $CDCl_3$): δ = 8.03–8.01 (m, 1H), 7.56 (ddd, *J* = 7.6, 1.4, 0.7 Hz, 1H), 7.41–7.40 (m, 1H), 7.37–7.34 (m, 1H), 7.33–7.32 (m, 2H), 7.30–7.29 (m, 1H), 7.23–7.21 (m, 1H), 6.56 (d, *J* = 0.7 Hz, 1H), 5.30 (s, 1H), 4.22–4.17 (m, 1H), 4.20 (d, *J* = 14.6 Hz, 1H), 4.02 (d, *J* = 14.4 Hz, 1H), 3.18 (s, 3H), 1.91–1.20 (m, 10H). $^{13}$C NMR (100 MHz, $CDCl_3$): δ = 153.8, 139.9, 137.6, 137.1, 135.2, 130.5, 129.0, 128.7, 127.5, 125.5, 125.1, 123.8, 121.1, 114.0, 111.4, 58.2, 56.1, 44.6, 40.8, 32.7, 32.6, 25.2, 24.7. FT-IR (ATR) $\nu_{max}$/cm$^{-1}$ 3337, 2940, 2854, 1599, 1454, 1170, 1061. HRMS (ESI$^+$): m/z: Calcd. for $C_{24}H_{28}ClN_6O_2S$ [M+H]$^+$: 499.1677; Found: 499.1681.

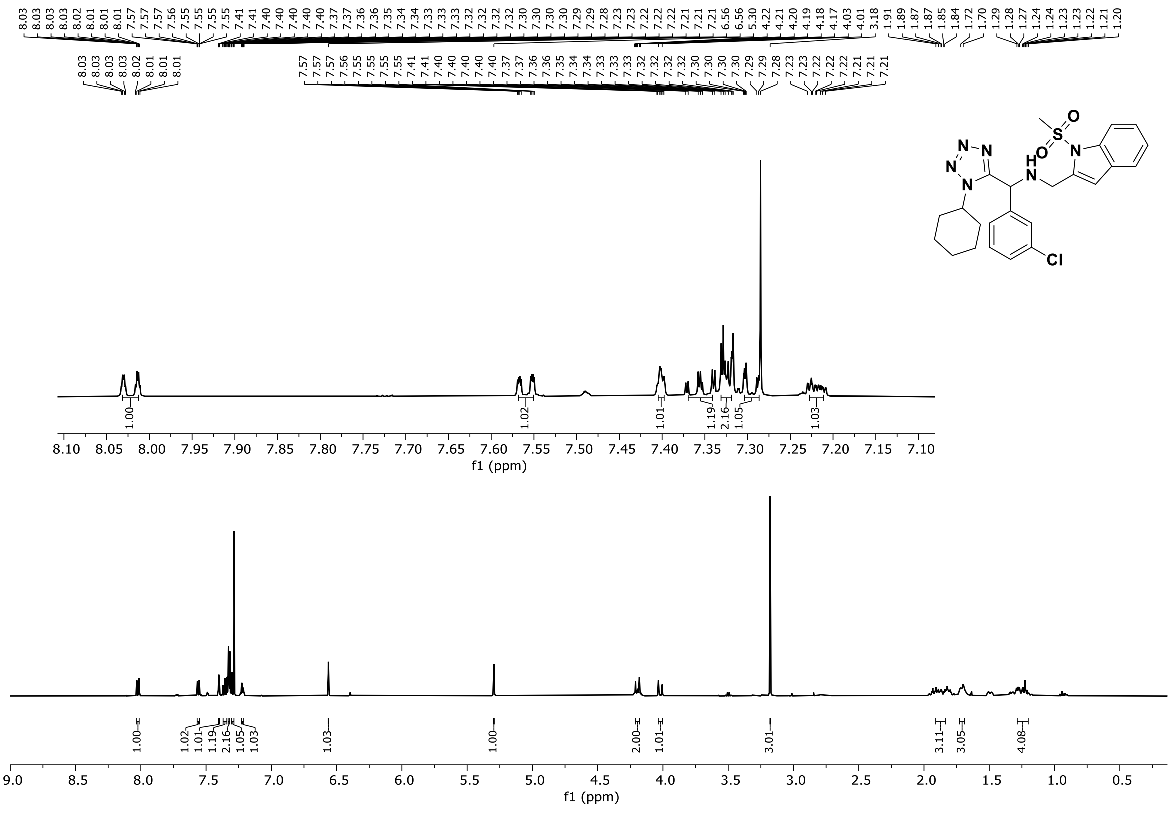


$^1$H NMR spectra of the compound **18h**

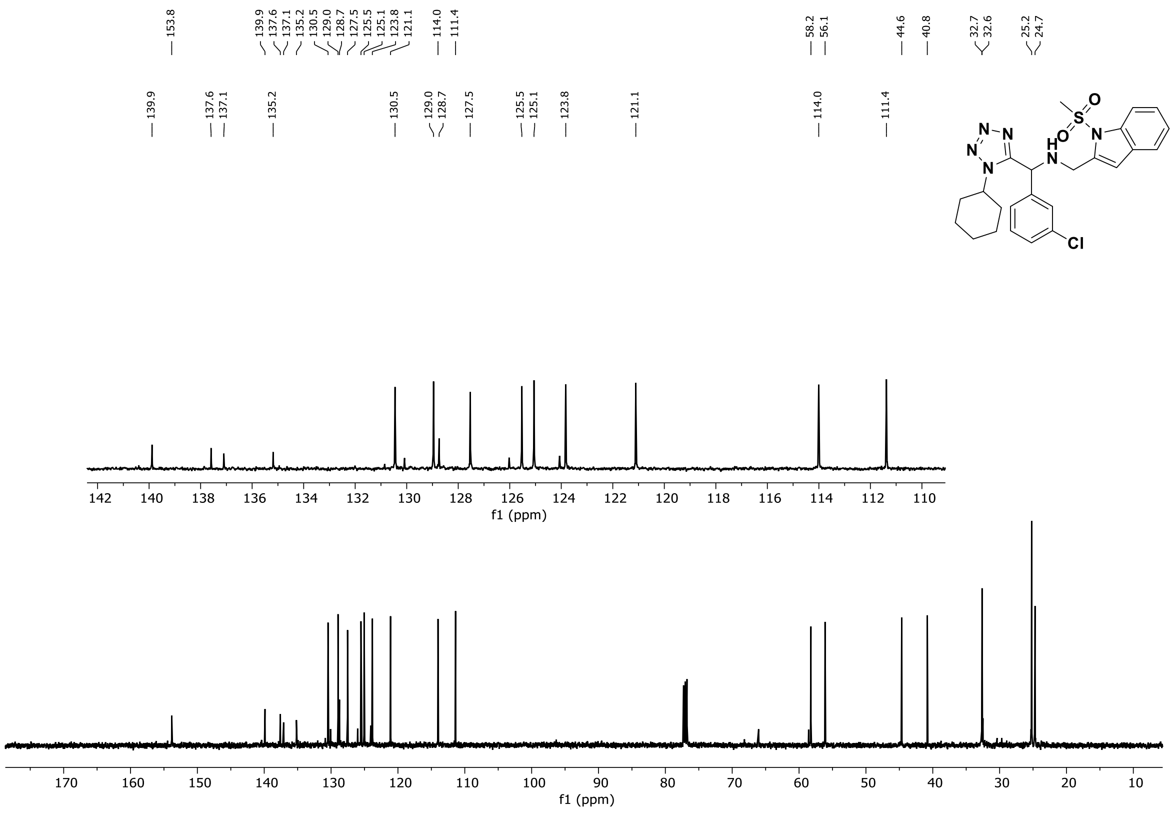


$^{13}$C NMR spectra of the compound **18h**

1-(1-(*tert*-Butyl)-1*H*-tetrazol-5-yl)-1-(2-chlorophenyl)-*N*-((1-(methylsulfonyl)-1*H*-indol-2-yl)methyl)methanamine (**18i)**

Based on the GP, propargylamine (23.3 µL, 0.36 mmol), 2-chlorobenzaldehyde (41.0 µL, 0.36 mmol), $TMSN_3$ (58.0 µL, 0.43 mmol), *tert*-butyl isocyanide (41.0 µL, 0.36 mmol), *N*-(2-iodophenyl)methanesulfonamide (129.4 mg, 0.43 mmol), $(PPh_3)_2PdCl_2$ (25.5 mg, 0.04 mmol) and CuI (5.5 mg, 0.03 mmol) were used and **18i** was obtained as an ambar oil (63.5 mg, 37%). $R_f$ = 0.43 (Hexane-AcOEt 7:3 v/v); $^1$H NMR (400 MHz, $CDCl_3$): δ = 7.99 (d, *J* = 8.4 Hz, 1H), 7.49 (d, *J* = 7.4 Hz, 1H), 7.41–7.39 (m, 1H), 7.31–7.27 (m, 1H), 7.25–7.21 (m, 4H), 6.50 (s, 1H), 5.98 (s, 1H), 4.26 (d, *J* = 13.9 Hz, 1H), 4.00 (d, *J* = 13.9 Hz, 1H), 3.24 (s, 3H), 1.60 (s, 9H). $^{13}$C NMR (100 MHz, $CDCl_3$): δ = 154.7, 138.6, 137.2, 136.5, 133.3, 130.4, 130.1, 129.4, 128.8, 128.1, 124.9, 123.6, 121.0, 114.1, 110.5, 61.9, 54.2, 45.1, 41.4, 30.0. FT-IR (ATR) $\nu_{max}$/cm$^{-1}$ 3331, 2997, 2921, 1593, 1456, 1364, 1172, 1063. HRMS (ESI$^+$): m/z: Calcd. for $C_{22}H_{26}ClN_6O_2S$ $[M+H]^+$: 473.1521; Found: 473.1517.

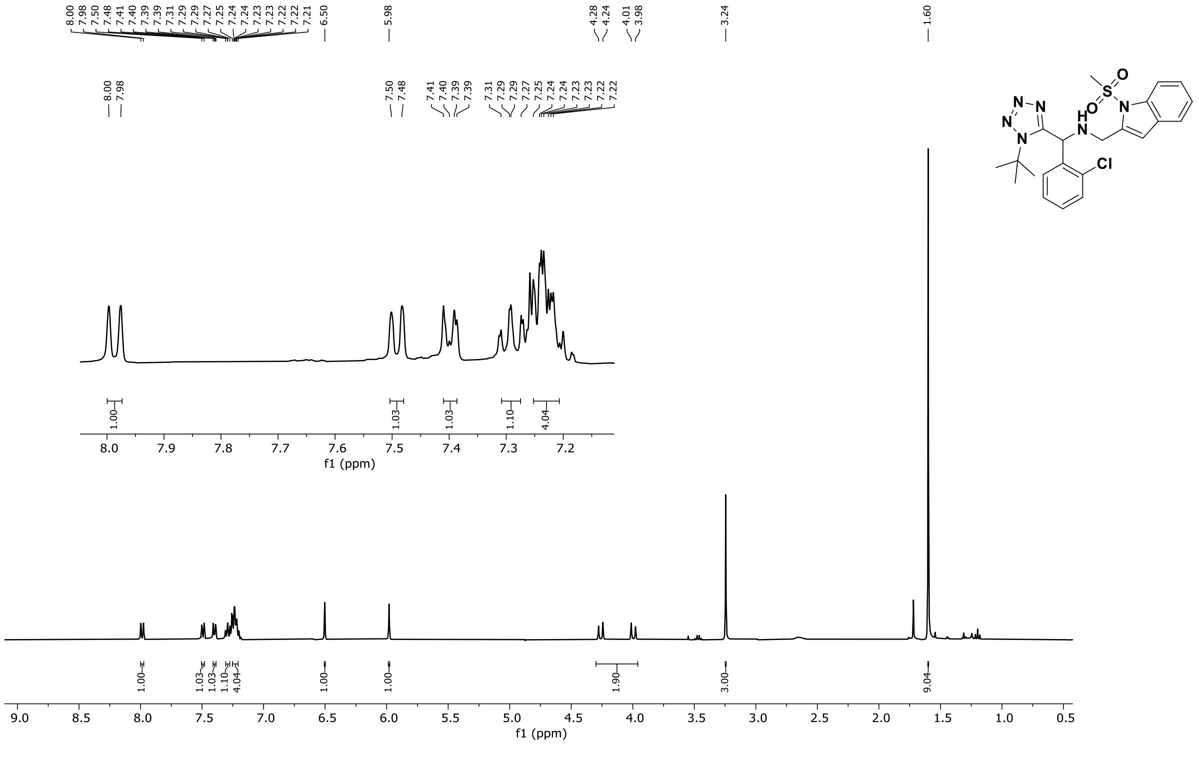


$^1$H NMR spectra of the compound **18i**

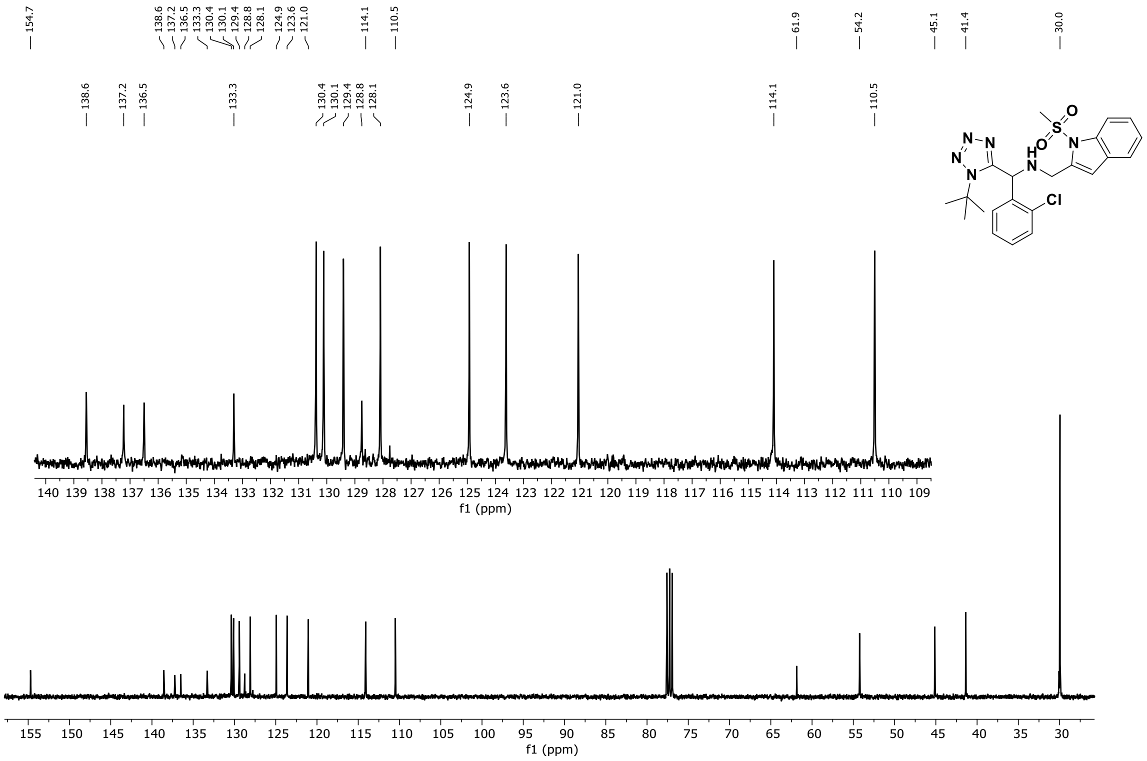


$^{13}$C NMR spectra of the compound **18i**

1-(1-Cyclohexyl-1*H*-tetrazol-5-yl)-*N*-((1-(methylsulfonyl)-1*H*-indol-2-yl)methyl)-1-phenylmethanamine (**18j)**

Based on the GP, propargylamine (23.3 µL, 0.36 mmol), benzaldehyde (37.0 µL, 0.36 mmol), $TMSN_3$ (58.0 µL, 0.43 mmol), cyclohexyl isocyanide (45.1 µL, 0.36 mmol), *N*-(2-iodophenyl)methanesulfonamide (129.4 mg, 0.43 mmol), $(PPh_3)_2PdCl_2$ (25.5 mg, 0.04 mmol) and CuI (5.5 mg, 0.03 mmol) were used and **18j** was obtained as a beige solid (55.6 mg, 33%). mp = 76–78 °C. $R_f$ = 0.40 (Hexane-AcOEt 7:3 v/v); $^1H$ NMR (400 MHz, $CDCl_3$): δ = 8.03 (dq, *J* = 8.4, 0.8 Hz, 1H), 7.55 (ddd, *J* = 7.6, 1.4, 0.7 Hz, 1H), 7.38–7.37 (m, 2H), 7.35–7.33 (m, 3H), 7.29–7.28 (m, 2H), 6.56 (s, 1H), 5.30 (s, 1H), 4.24 (d, *J* = 14.4 Hz, 1H), 4.17–4.12 (m, 1H), 4.03 (d, *J* = 14.4 Hz, 1H), 3.20 (s, 3H), 2.76 (bs, 1H), 1.94–1.22 (m, 10H). $^{13}C$ NMR (100 MHz, $CDCl_3$): δ = 154.4, 138.0, 137.9, 137.1, 129.2, 128.7, 127.3, 124.9, 123.7, 121.0, 114.0, 111.0, 58.1, 56.7, 44.6, 40.9, 32.6, 32.5, 25.2, 25.2, 24.7. FT-IR (ATR) $\nu_{max}/cm^{-1}$ 3312, 2923, 2858, 1587, 1458, 1358, 1168, 1062. HRMS ($ESI^+$): m/z: Calcd. for $C_{24}H_{29}N_6O_2S[M+H]^+$: 465.2067; Found: 465.2061.

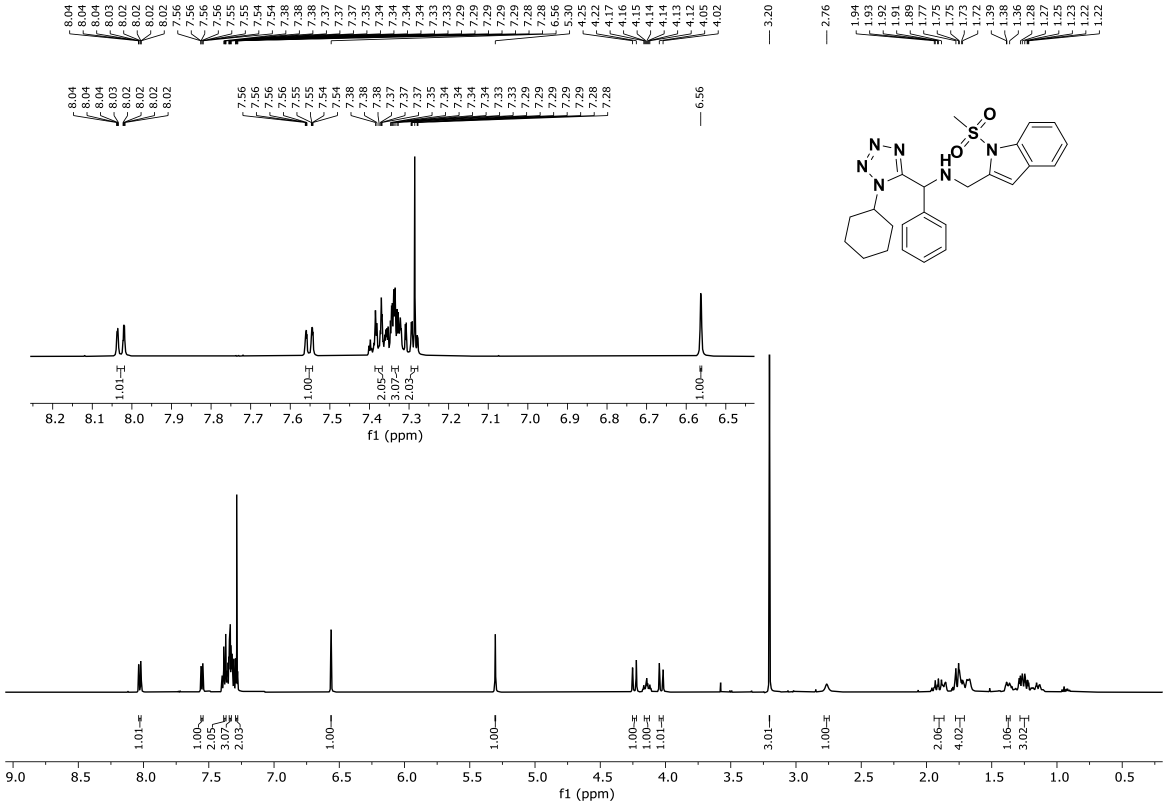


$^1H$ NMR spectra of the compound **18j**

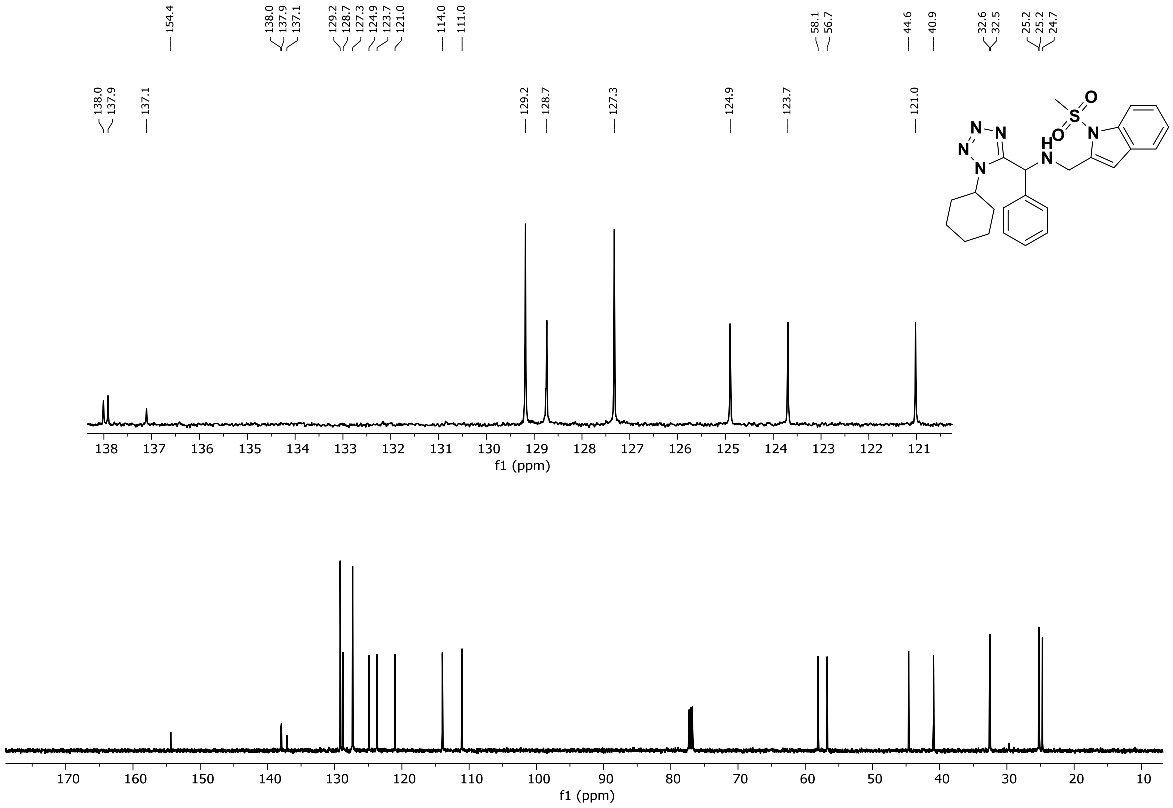


$^{13}$C NMR spectra of the compound **18j**

1-(2-Chlorophenyl)-1-(1-cyclohexyl-1*H*-tetrazol-5-yl)-*N*-((1-(methylsulfonyl)-1*H*-indol-2-yl)methyl)methanamine (**18k)**

Based on the GP, propargylamine (23.3 µL, 0.36 mmol), 2-chlorobenzaldehyde (41.0 µL, 0.36 mmol), $TMSN_3$ (58.0 µL, 0.43 mmol), cyclohexyl isocyanide (45.1 µL, 0.36 mmol), *N*-(2-iodophenyl)methanesulfonamide (129.4 mg, 0.43 mmol), $(PPh_3)_2PdCl_2$ (25.5 mg, 0.04 mmol) and CuI (5.5 mg, 0.03 mmol) were used and **18k** was obtained as an yellow oil (72.4 mg, 40%). $R_f$ = 0.36 (Hexane-AcOEt 7:3 v/v); $^1$H NMR (400 MHz, $CDCl_3$): δ = 8.00 (dq, *J* = 8.3, 0.9 Hz, 1H), 7.52 (ddd, *J* = 7.7, 1.4, 0.8 Hz, 1H), 7.43–7.40 (m, 2H), 7.33–7.26 (m, 4H), 6.53 (d, *J* = 0.8 Hz, 1H), 5.77 (s, 1H), 4.20 (d, *J* = 14.4 Hz, 1H), 4.11 (d, *J* = 15.0 Hz, 1H), 4.09–4.05 (m, 1H), 3.21 (s, 3H), 2.80 (bs, 1H), 2.04–1.19 (m, 10H). $^{13}$C NMR (100 MHz, $CDCl_3$): δ = 154.2, 138.1, 137.0, 135.6, 133.0, 130.0, 129.8, 129.3, 128.6, 128.0, 124.8, 123.5, 120.8, 113.9, 110.6, 57.9, 52.2, 44.3, 40.9, 32.6, 32.5, 25.1, 24.7, 24.6. FT-IR (ATR) $\nu_{max}$/cm$^{-1}$ 3334, 2948, 2856, 1590, 1454, 1170, 1057. HRMS (ESI$^+$): m/z: Calcd. for $C_{24}H_{28}ClN_6O_2S$ [M+H]$^+$: 499.1677; Found: 499.1679.

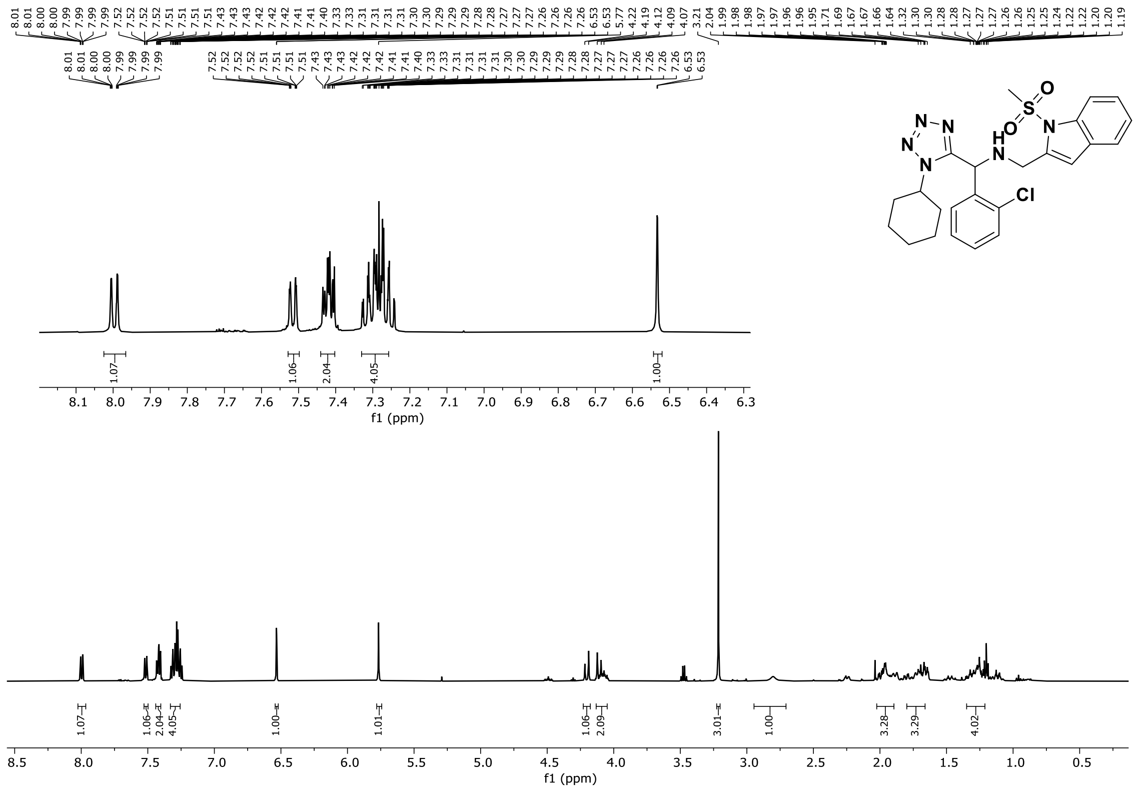


$^1$H NMR spectra of the compound **18k**

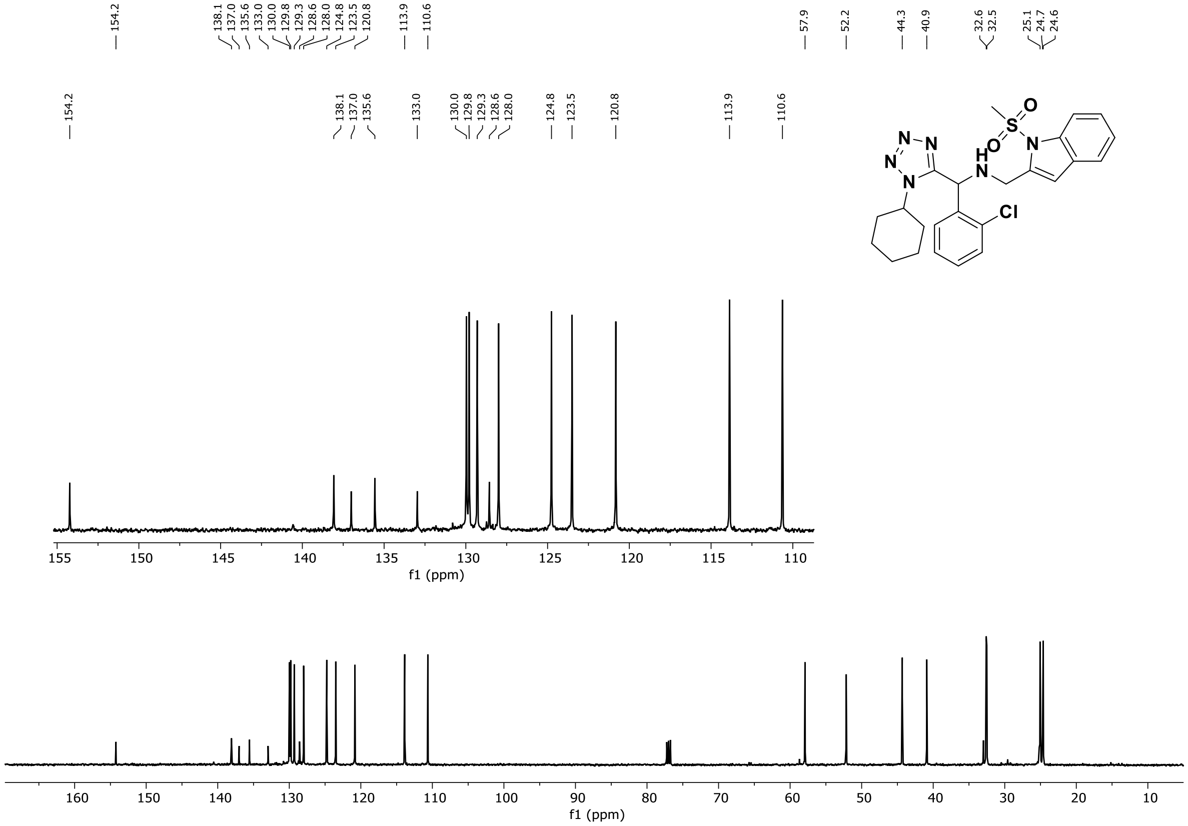

$^{13}$C NMR spectra of the compound **18k**

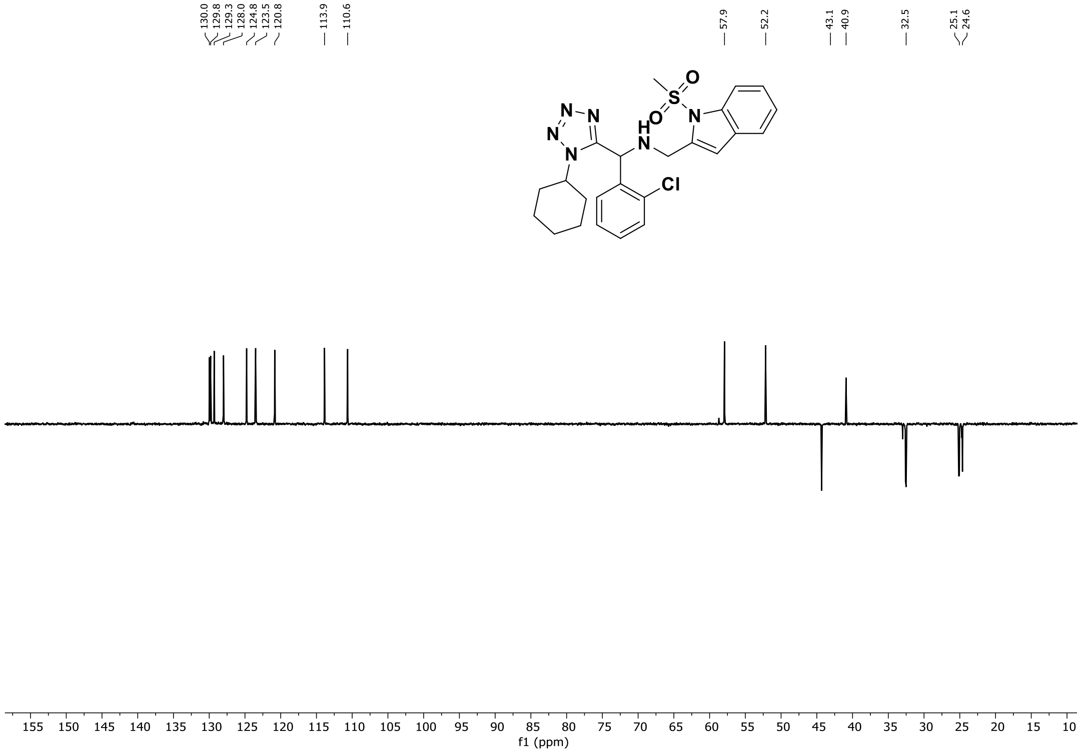

DEPT-135 NMR spectra of the compound **18k**

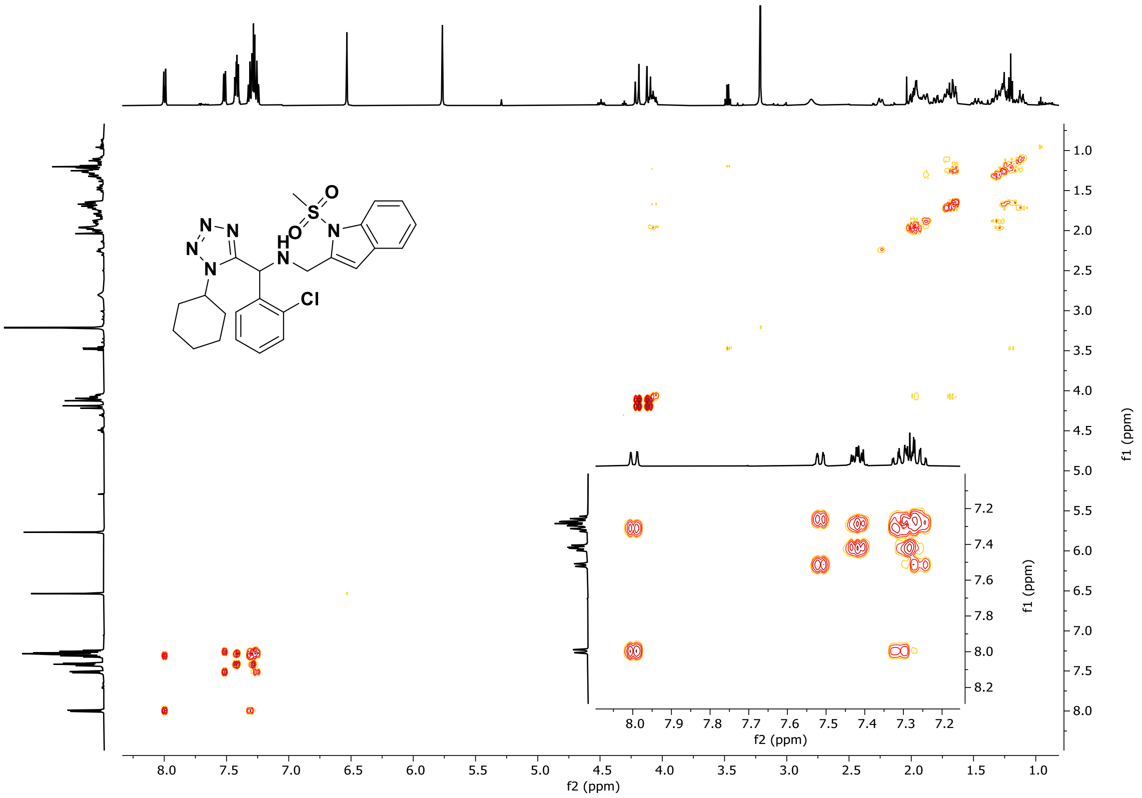

COSY NMR spectra of the compound **18k**

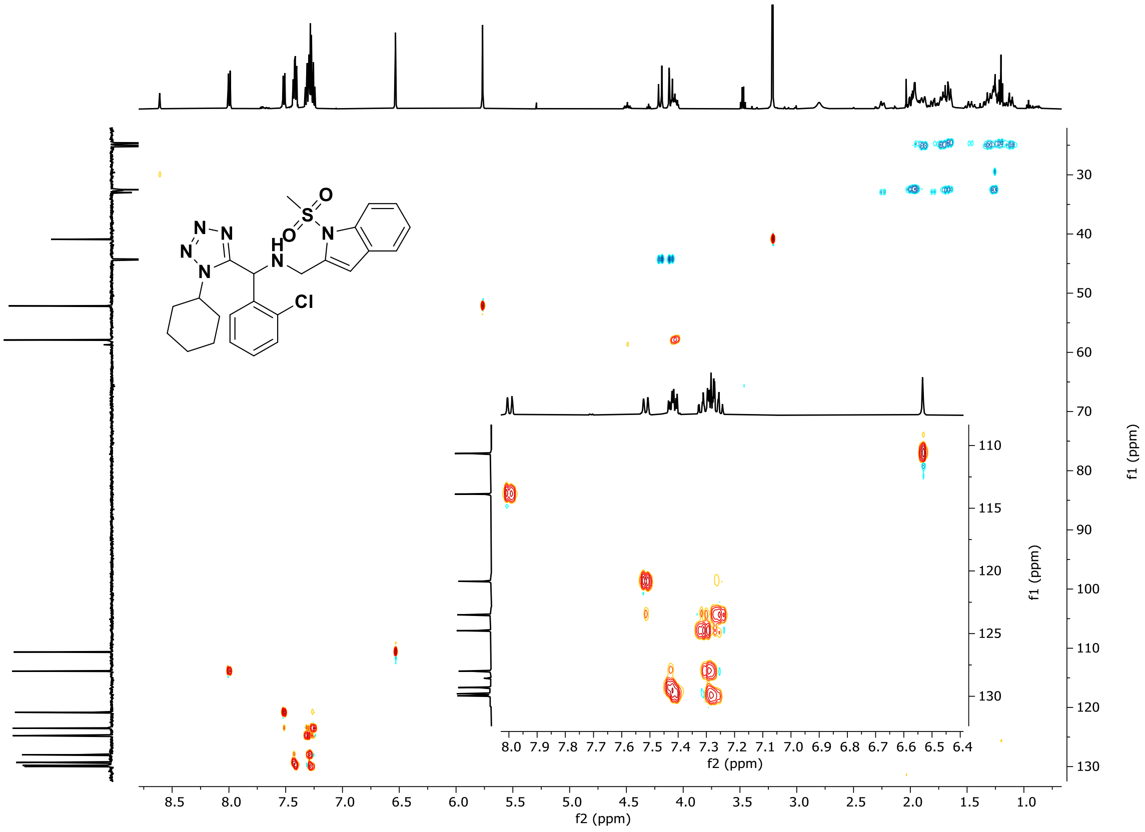

HSQC NMR spectra of the compound **18k**

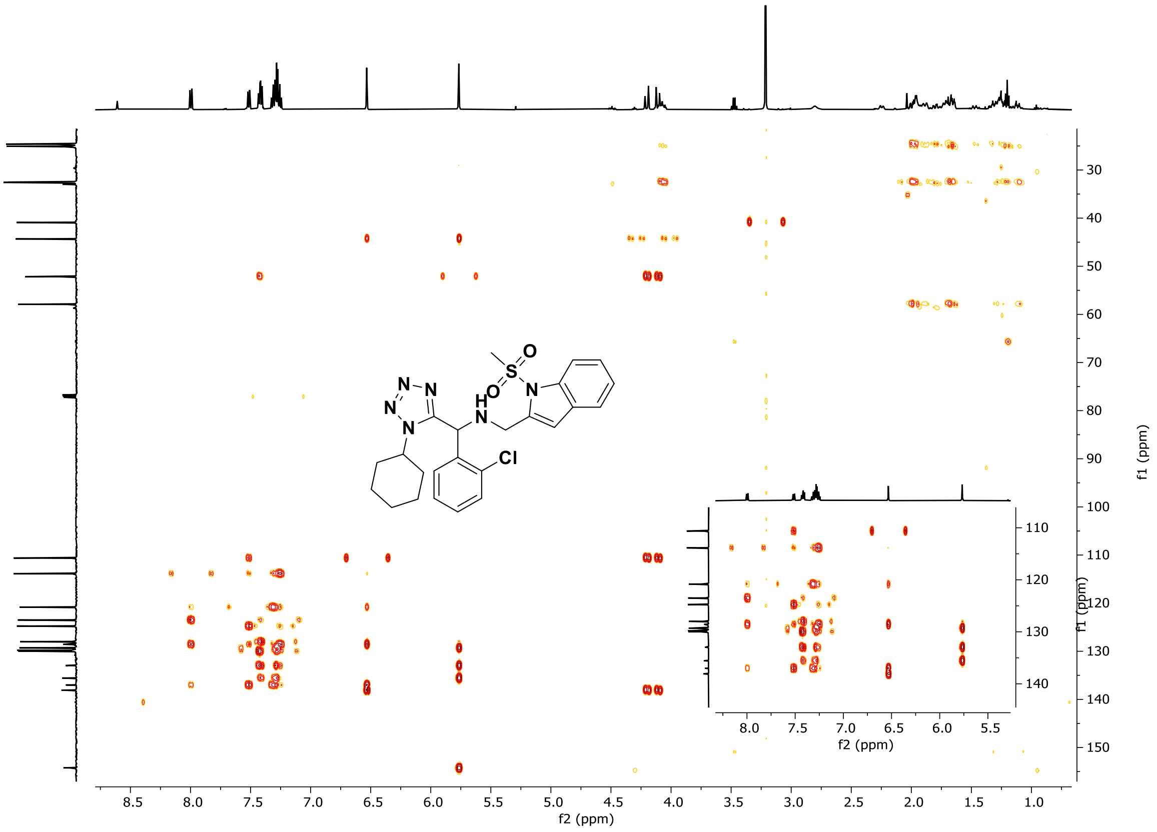


HMBC NMR spectra of the compound **18k**

1-(1-(*tert*-Butyl)-1*H*-tetrazol-5-yl)-*N*-((1-(methylsulfonyl)-1*H*-indol-2-yl)methyl)-1-phenylmethanamine (**18l)**

Based on the GP, propargylamine (23.3 µL, 0.36 mmol), benzaldehyde (37.0 µL, 0.36 mmol), $TMSN_3$ (58.0 µL, 0.43 mmol), *tert*-butyl isocyanide (41.0 µL, 0.36 mmol), *N*-(2-iodophenyl)methanesulfonamide (129.4 mg, 0.43 mmol), $(PPh_3)_2PdCl_2$ (25.5 mg, 0.04 mmol) and CuI (5.5 mg, 0.03 mmol) were used and **18l** was obtained as a yellow solid (55.7 mg, 35%). mp = 138–140 °C. $R_f$ = 0.43 (Hexane-AcOEt 7:3 v/v); $^1$H NMR (400 MHz, $CDCl_3$): δ = 8.00 (dd, *J* = 8.3, 0.9 Hz, 1H), 7.50 (ddd, *J* = 7.6, 1.4, 0.7 Hz, 1H), 7.36–7.34(m, 1H), 7.33–7.27 (m, 5H), 7.25–7.22 (m, 1H), 6.49 (d, *J* = 0.8 Hz, 1H), 5.52 (s, 1H), 4.14 (d, *J* = 13.7 Hz, 1H), 3.98 (d, *J* = 13.7 Hz, 1H), 3.23 (s, 3H), 2.63 (bs, 1H), 1.61 (s, 9H). $^{13}$C NMR (100 MHz, $CDCl_3$): δ = 155.0, 138.5, 137.0, 129.0, 128.5, 128.5, 128.0, 124.6, 123.4, 120.8, 113.8, 109.9, 61.4, 59.0, 45.0, 41.0, 29.9. FT-IR (ATR) $\nu_{max}$/cm$^{-1}$ 3302, 2952, 1597, 1352, 1163, 1051. HRMS (ESI$^+$): m/z: Calcd. for $C_{22}H_{27}N_6O_2S[M+H]^+$: 439.1911; Found: 439.1921.

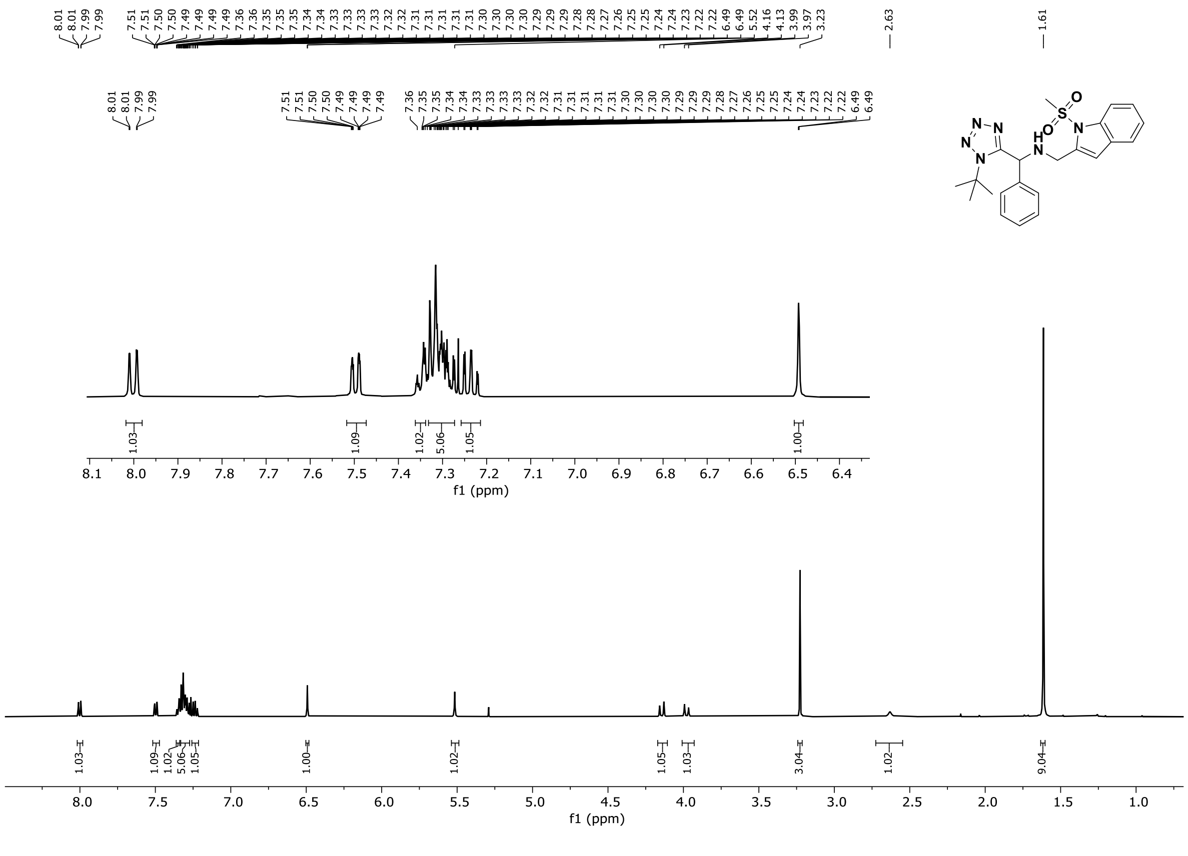


$^1$H NMR spectra of the compound **18l**

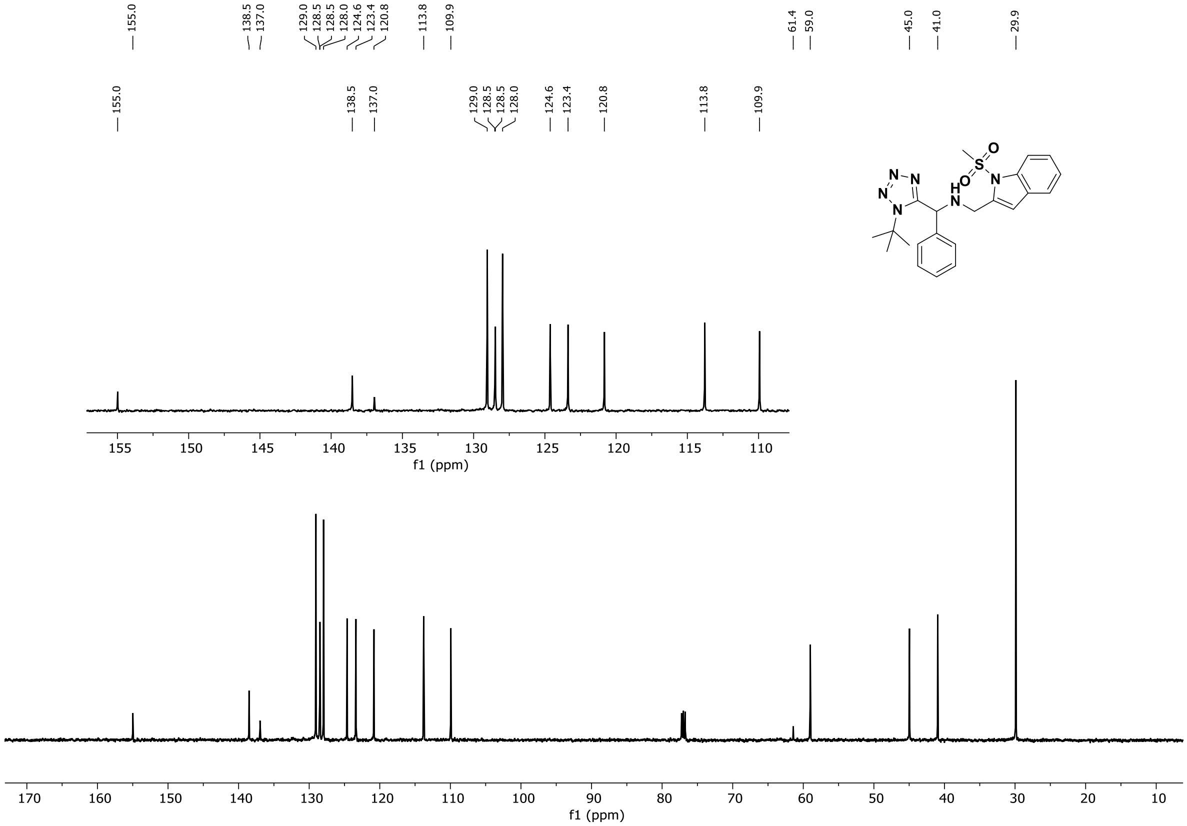


$^{13}C$ NMR spectra of the compound **18l**

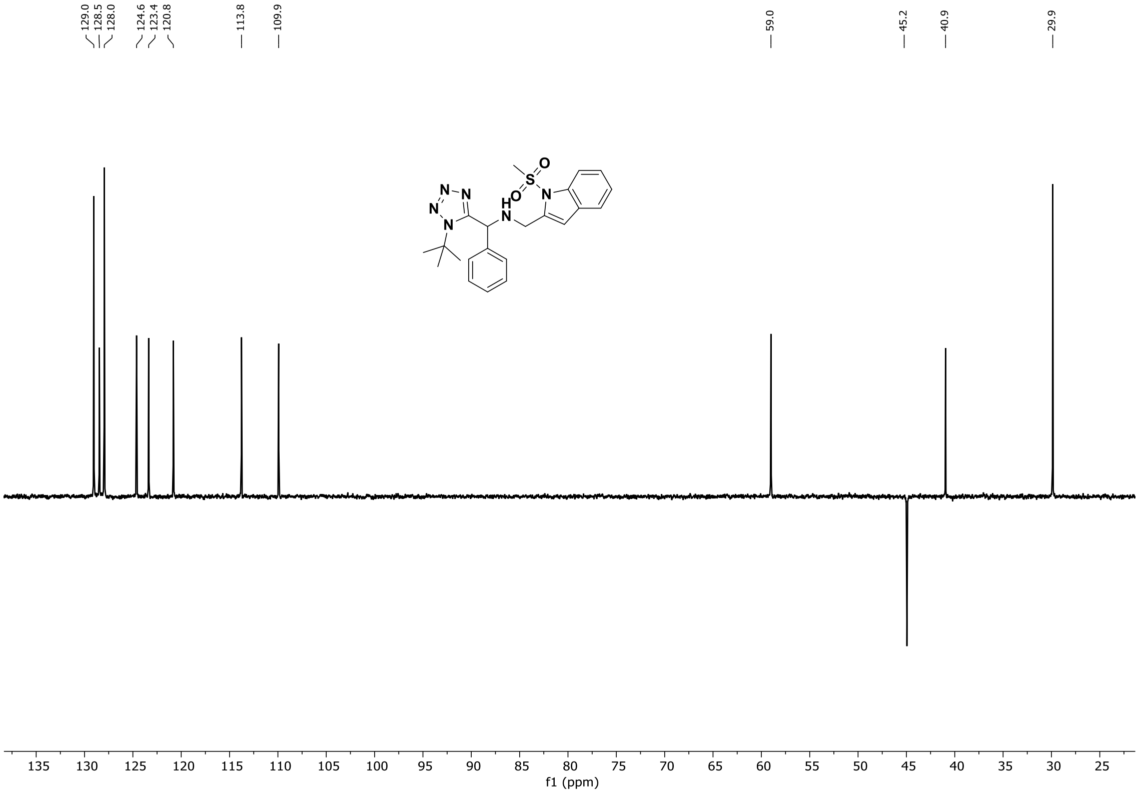


DEPT-135 NMR spectra of the compound **18l**

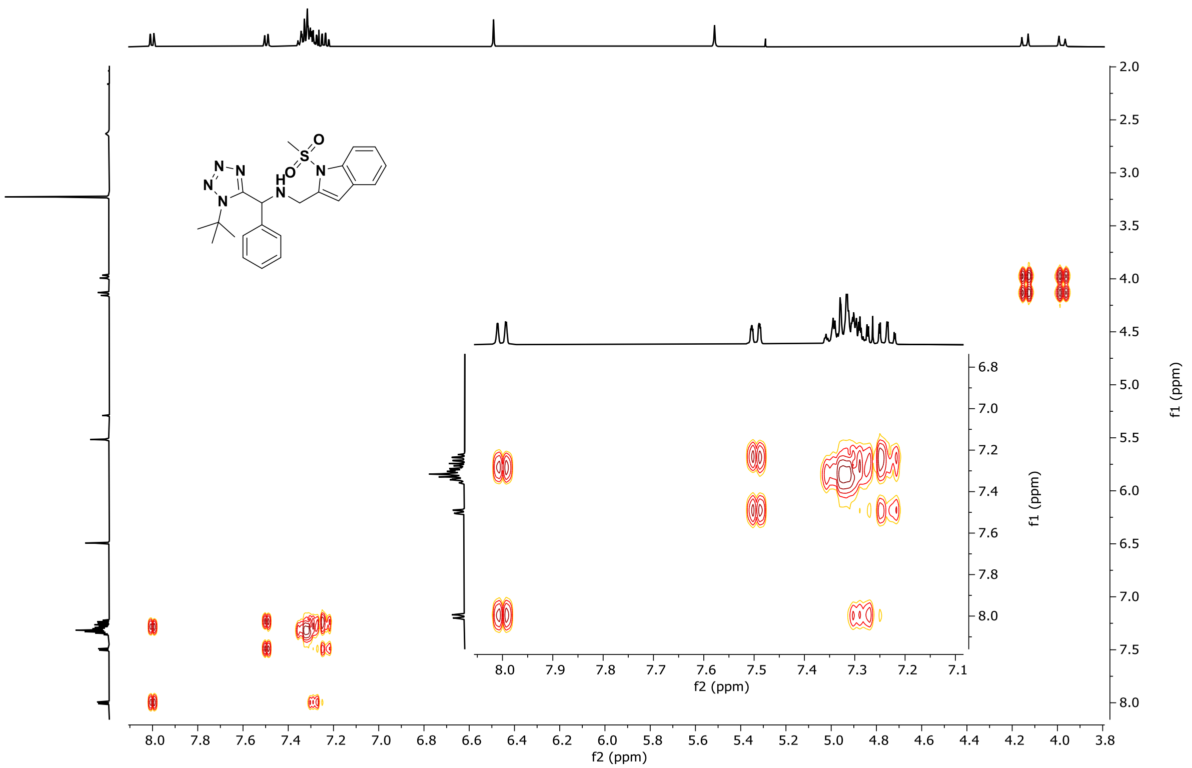


COSY NMR spectra of the compound **18l**

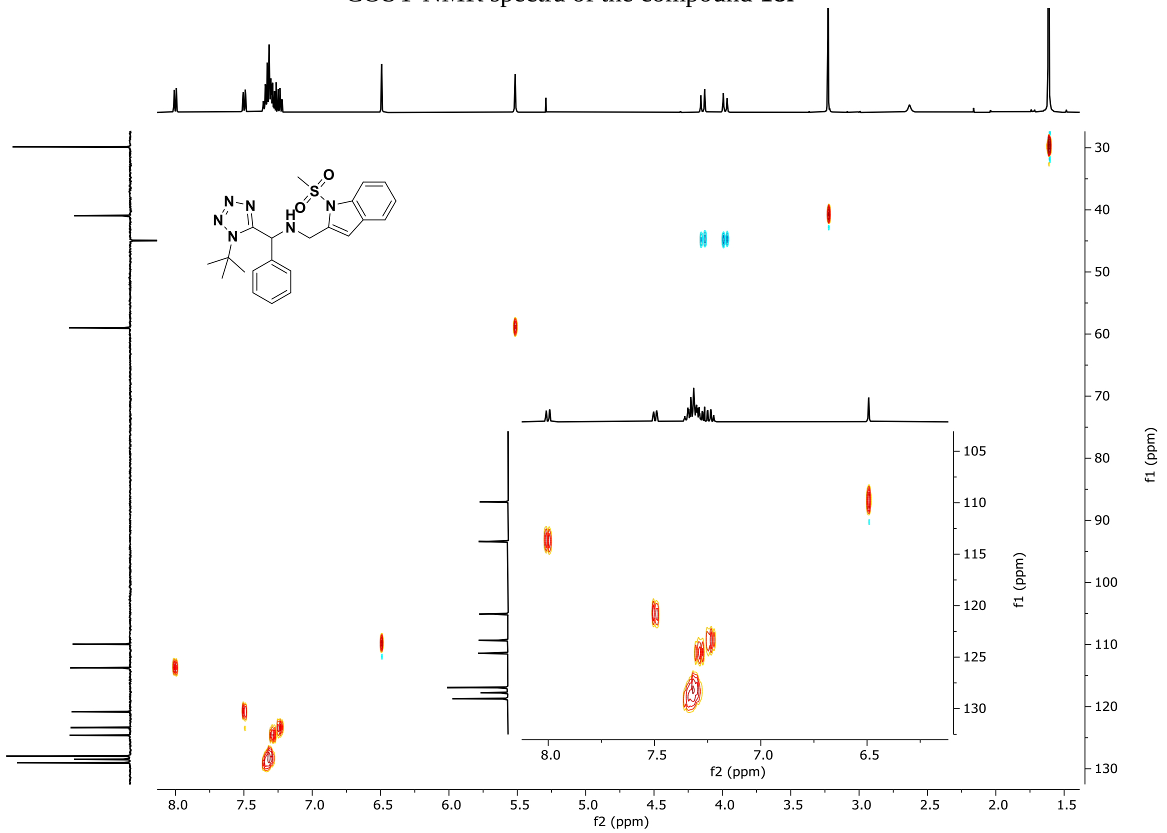


HSQC NMR spectra of the compound **18l**

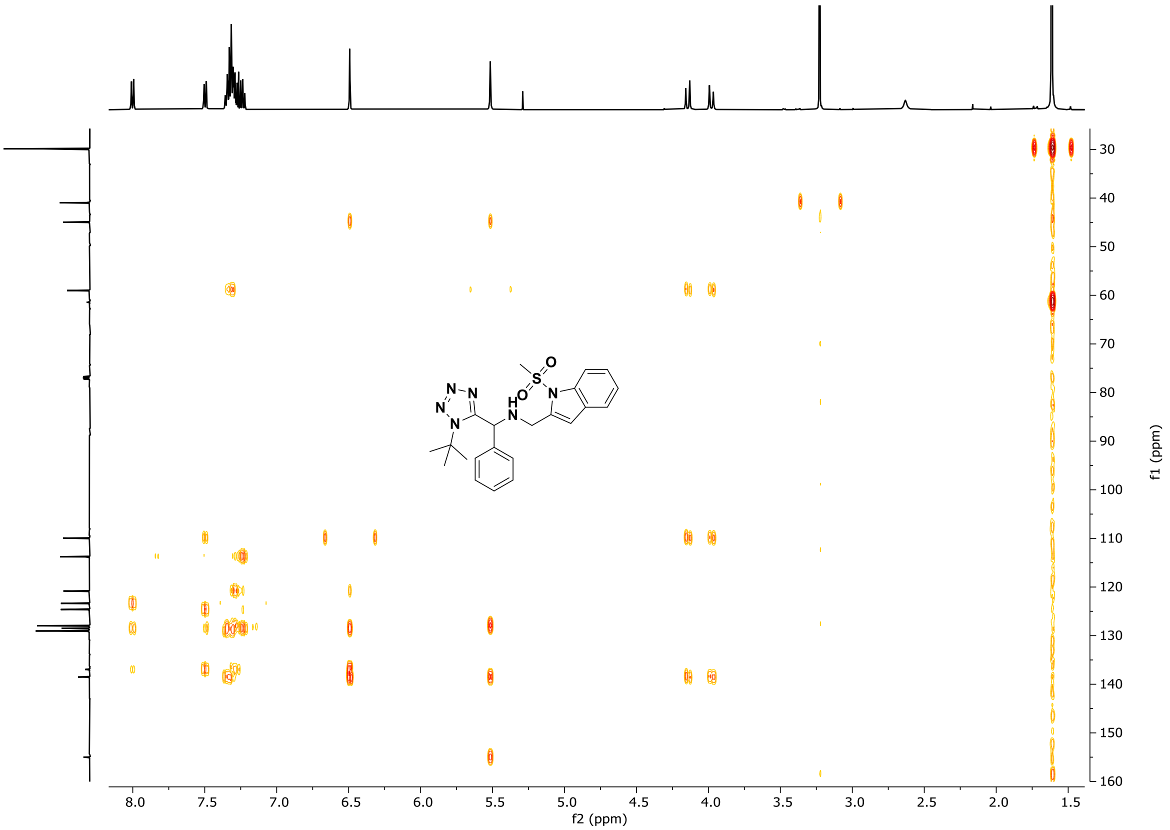

HMBC NMR spectra of the compound **18l**

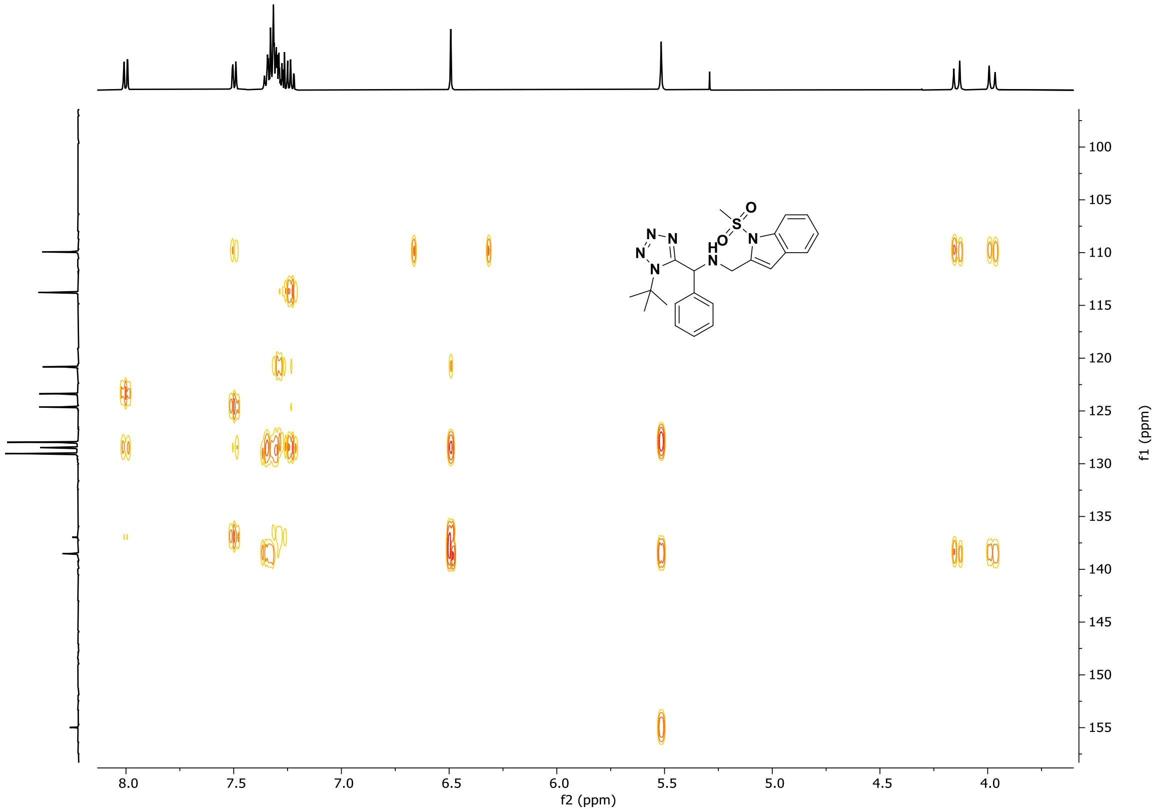

HMBC NMR spectra of the compound **18l** (expansion)

1-(1-(*tert*-Butyl)-1*H*-tetrazol-5-yl)-1-(4-methoxyphenyl)-*N*-((1-(methylsulfonyl)-1*H*-indol-2-yl)methyl)methanamine (**18m)**

Based on the GP, propargylamine (23.3 µL, 0.36 mmol), 4-methoxybenzaldehyde (44.1 µL, 0.36 mmol), $TMSN_3$ (58.0 µL, 0.43 mmol), *tert*-butyl isocyanide (41.0 µL, 0.36 mmol), *N*-(2-iodophenyl)methanesulfonamide (129.4 mg, 0.43 mmol), $(PPh_3)_2PdCl_2$ (25.5 mg, 0.04 mmol) and CuI (5.5 mg, 0.03 mmol) were used and **18m** was obtained as a yellow semisolid (30.6 mg, 18%). $R_f$ = 0.36 (Hexane-AcOEt 7:3 v/v); $^1$H NMR (400 MHz, $CDCl_3$): δ = 8.00 (d, *J* = 8.2 Hz, 1H), 7.50 (d, *J* = 7.4 Hz, 1H), 7.31–7.23 (m, 2H), 7.21 (d, *J* = 8.7 Hz, 2H), 6.84 (d, *J* = 8.7 Hz, 2H), 6.49 (s, 1H), 5.46 (s, 1H), 4.11 (d, *J* = 13.7 Hz, 1H), 3.95 (d, *J* = 13.7 Hz, 1H), 3.77 (s, 3H), 3.24 (s, 3H), 1.61 (s, 9H). $^{13}$C NMR (100 MHz, $CDCl_3$): δ = 159.8, 155.5, 138.9, 137.2, 130.9, 129.5, 128.8, 124.9, 123.7, 121.1, 114.6, 114.1, 110.2, 61.7, 58.7, 55.5, 45.2, 41.3, 30.2. FT-IR (ATR) $\nu_{max}$/cm$^{-1}$ 3316, 2923, 2846, 1611, 1456, 1364, 1255, 1172, 1059. HRMS (ESI$^+$): m/z: Calcd. for $C_{23}H_{29}N_6O_3S$ [M+H]$^+$: 469.2016; Found: 469.2018.

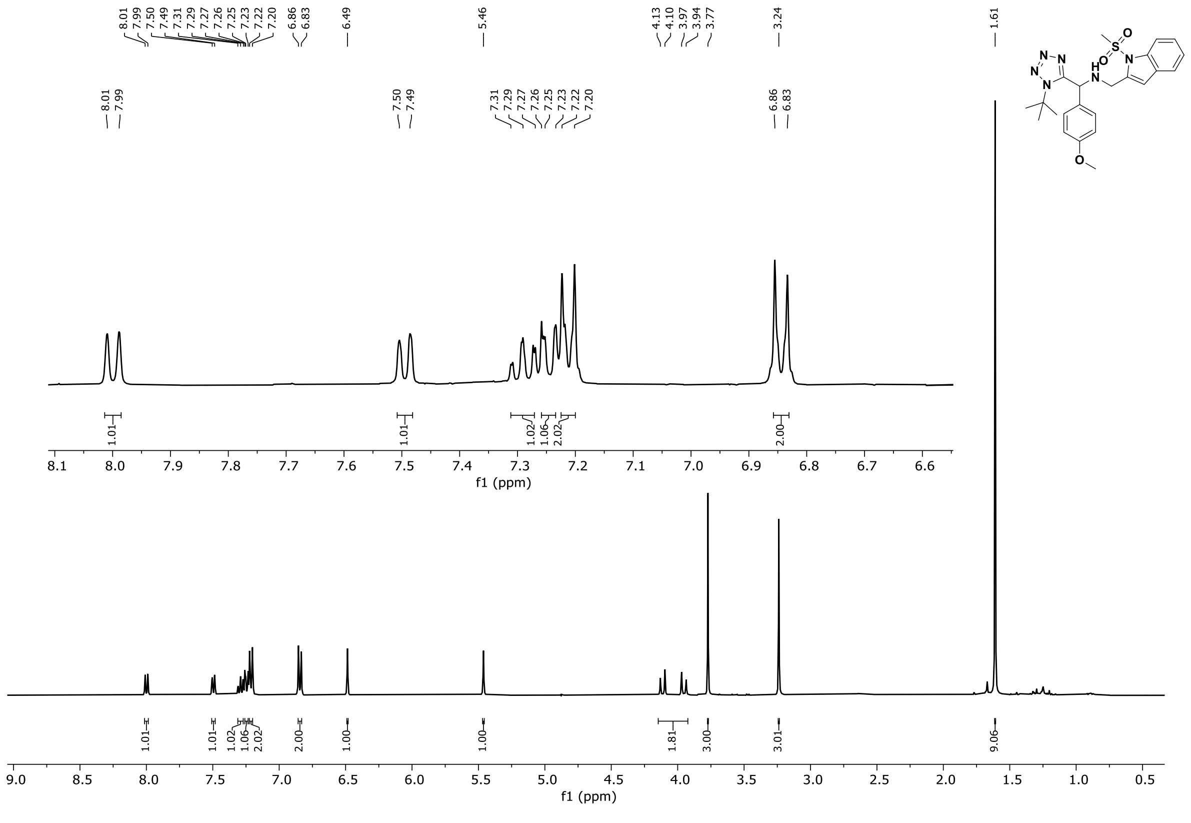


$^1$H NMR spectra of the compound **18m**

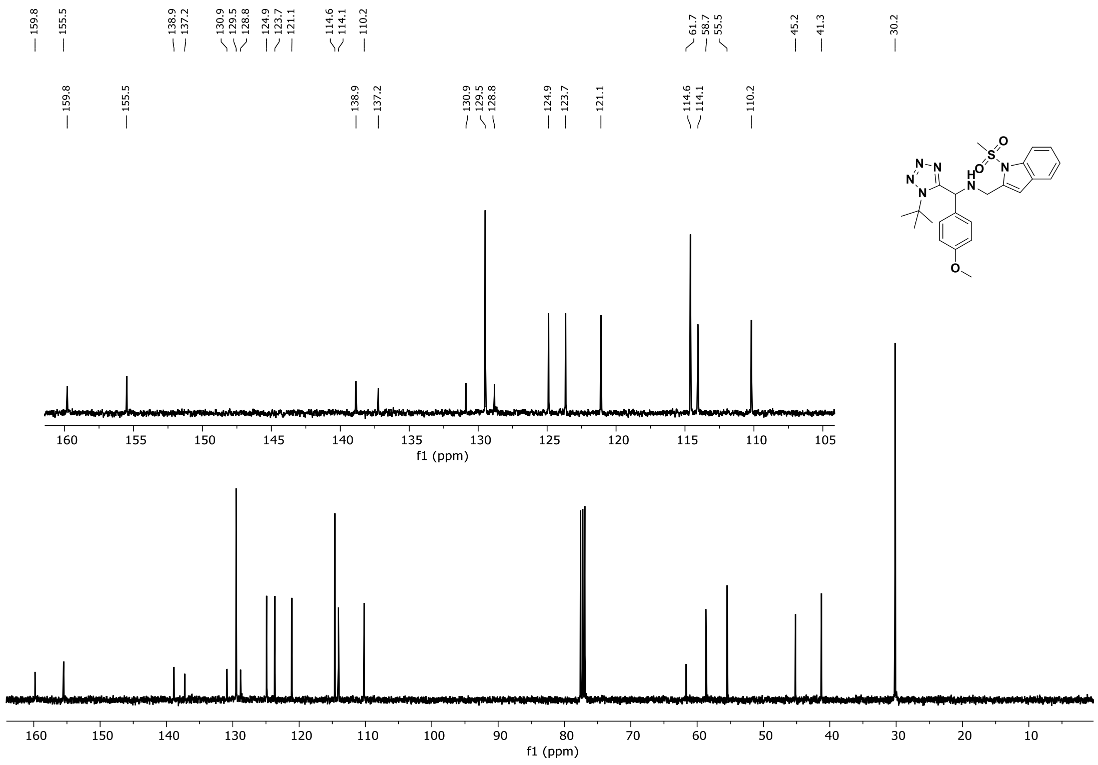


$^{13}$C NMR spectra of the compound **18m**

1-(1-(*tert*-butyl)-1*H*-tetrazol-5-yl)-1-(4-methoxyphenyl)-*N*-((1-(methylsulfonyl)-1*H*-indol-2-yl)methyl)methanamine (**18n)**

Based on the GP, propargylamine (23.3 µL, 0.36 mmol), 4-methoxybenzaldehyde (44.1 µL, 0.36 mmol), $TMSN_3$ (58.0 µL, 0.43 mmol), cyclohexyl isocyanide (45.1 µL, 0.36 mmol), *N*-(2-iodophenyl)methanesulfonamide (129.4 mg, 0.43 mmol), $(PPh_3)_2PdCl_2$ (25.5 mg, 0.04 mmol) and CuI (5.5 mg, 0.03 mmol) were used and **18n** was obtained as an yellow oil (28.7 mg, 16%). $R_f$ = 0.30 (Hexane-AcOEt 7:3 v/v); $^1$H NMR (400 MHz, $CDCl_3$): δ = 8.00 (d, *J* = 8.2 Hz, 1H), 7.52 (d, *J* = 7.2 Hz, 1H), 7.34–7.30 (m, 1H), 7.30–7.25 (m, 1H), 7.21 (d, *J* = 8.7 Hz, 2H), 6.86 (d, *J* = 8.7 Hz, 2H), 6.52 (s, 1H), 5.20 (s, 1H), 4.19 (d, *J* = 14.3 Hz, 1H), 4.14–4.06 (m, 1H), 3.99 (d, *J* = 14.3 Hz, 1H), 3.78 (s, 3H), 3.19 (s, 3H), 1.96–1.11 (m, 10H). $^{13}$C NMR (100 MHz, $CDCl_3$): δ = 160.0, 154.8, 138.3, 137.3, 130.1, 129.0, 128.8, 125.1, 123.9, 121.2, 114.7, 114.2, 111.1, 58.2, 56.4, 55.6, 44.8, 41.2, 32.8, 32.7, 25.5, 25.5, 24.9. FT-IR (ATR) $\nu_{max}$/cm$^{-1}$ 3314, 2982, 2958, 1564, 1462, 1169, 1054. HRMS (ESI$^+$): m/z: Calcd. for $C_{25}H_{30}N_6O_3S$ [M+H]$^+$: 494.2100; Found: 494.2098.

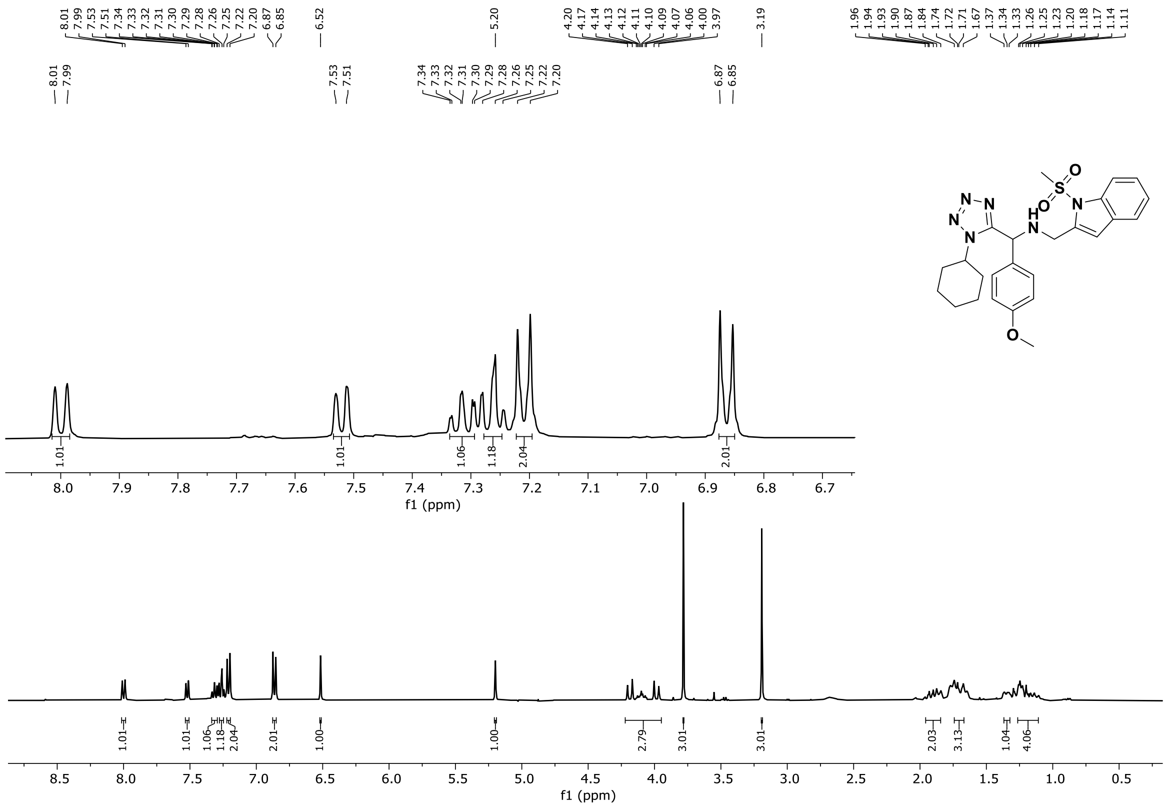


$^1$H NMR spectra of the compound **18n**

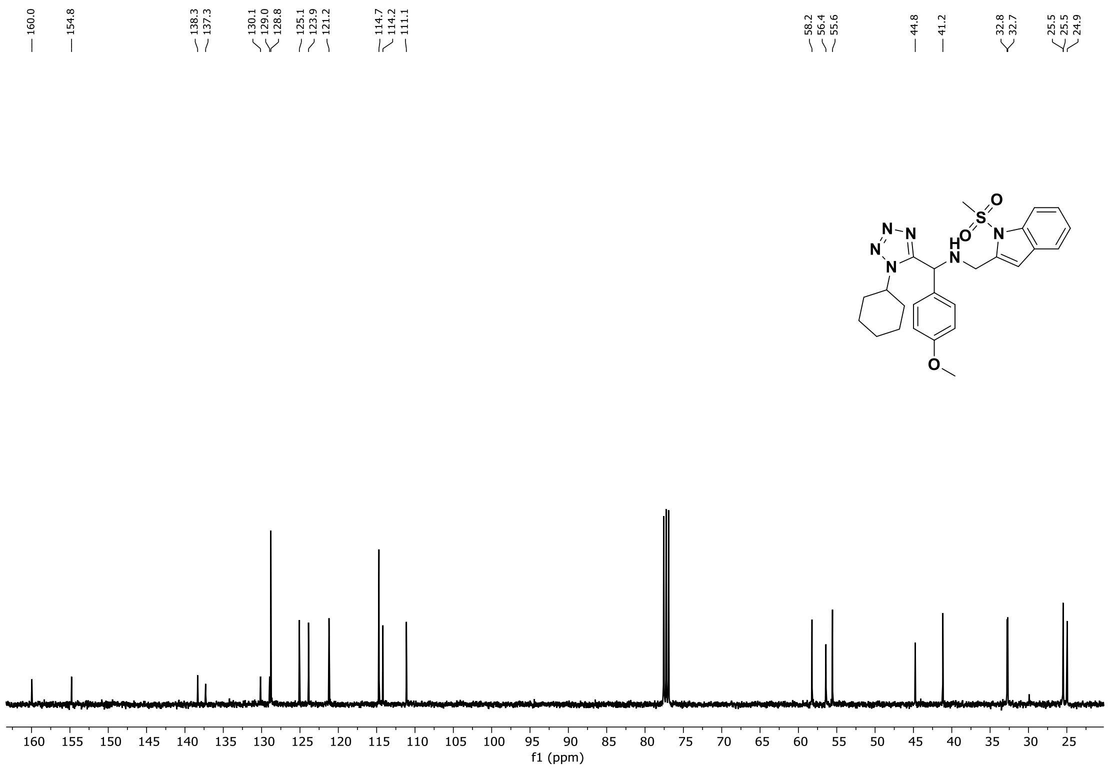

$^{13}$C NMR spectra of the compound **18n**

Biological evaluation of compounds **18a–j**

**Note:** The general procedure reported herein follows a methodology analogous to that described by the Cortés-García research group.[4]

***Cell lines.***

The breast tumor cell line MCF-7 was grown in Dulbecco's Modified Eagle's Medium (DMEM) (Invitrogen Corporation, Carlsbad, CA, United States) enriched with 5% fetal bovine serum (FBS). Medium change and passage were performed every 3 and 4 days, respectively. The MCF-7 cell line was kindly provided by Ph.D. Victor Treviño from Tecnológico de Monterrey.

***Cell proliferation analysis.***

Cell proliferation was quantified by violet crystal dye in 1× phosphate-buffered saline (PBS) (2.7 mM KCl, 1.8 mM $KH_2PO_4$, 136 mM NaCl, 10 mM $Na_2HPO_4$, pH 7.4). The treated cells were incubated in methanol for 15 min and washed two times with water. Cells were dyed with 0.1% crystal violet and washed three times with water, and finally, crystal violet was recovered with 10% acid acetic to be analyzed in microplate reader Multiskan GO Spectrophotometer (Thermo Scientific™, Ratastic, Finland).

$IC_{50}$ determination. The $IC_{50}$ was determined in Graph Pad Prism version 8.0 for Windows (Prism Software, La Jolla, California, USA, www.graphpad.com)

**References**


1. Debnath, S.; Mondal, S. *Tetrahedron Lett*. **2018**, *59*, 2260-2263. doi: 10.1016/j.tetlet.2018.04.081.
2. Buarque, C. D.; Salustiano, E. J.; Fraga, K. C.; Alves, B. R. M.; Costa, P. R. R. *Eur. J. Med. Chem*. **2014**, *78*, 190-197. doi: 10.1016/j.ejmech.2014.03.039.
3. Aguilar-Morales, C. M.; Alejandre-Castañeda, V.; Contreras-Celedón, C.; Ramírez-Díaz, M. I.; Islas-Jácome, A.; Meza-Carmen, V.; Chacón-García, L.; Cortés-García, C.J. *Org. Biomol. Chem.* **2024**, *22*, 7240–7244. doi: 10.1039/D4OB00995A
4. Aguilar-Morales, C. M.; Araujo-Huitrado, J. G.; López-Hernández, Y.; Contreras-Celedón, C.; Islas-Jácome, A.; Granados-López, A. J.; Solorio-Alvarado, C. R.; López, J. A.; Chacón-García, L.; Cortés-García, C.J. *Molecules* **2021**, *26*, 1–14. doi: 10.3390/molecules26206104
